# Stimuli‐responsive linkers and their application in molecular imaging

**DOI:** 10.1002/EXP.20230027

**Published:** 2024-01-18

**Authors:** Jing Wang, Meng Liu, Xinyue Zhang, Xinning Wang, Menghua Xiong, Dong Luo

**Affiliations:** ^1^ School of Biomedical Sciences and Engineering South China University of Technology Guangzhou P. R. China; ^2^ Department of Biomedical Engineering Case Western Reserve University Cleveland Ohio USA; ^3^ National Engineering Research Centre for Tissue Restoration and Reconstruction South China University of Technology Guangzhou P. R. China

**Keywords:** activatable imaging agent, biomarkers, molecular imaging, stimuli‐responsive linkers

## Abstract

Molecular imaging is a non‐invasive imaging method that is widely used for visualization and detection of biological events at cellular or molecular levels. Stimuli‐responsive linkers that can be selectively cleaved by specific biomarkers at desired sites to release or activate imaging agents are appealing tools to improve the specificity, sensitivity, and efficacy of molecular imaging. This review summarizes the recent advances of stimuli‐responsive linkers and their application in molecular imaging, highlighting the potential of these linkers in the design of activatable molecular imaging probes. It is hoped that this review could inspire more research interests in the development of responsive linkers and associated imaging applications.

## Graphical abstract

Stimuli‐responsive linkers are appealing tools to improve the specificity, sensitivity, and efficacy of molecular imaging. In this review, the recent examples and progress toward the development of stimuli‐responsive linkers and their application in molecular imaging are systematically summarized, highlighting the potential of these linkers in the design of activatable molecular imaging probes.

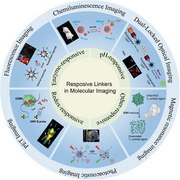

## 1 INTRODUCTION

Molecular imaging, which involves quantitative, non‐invasive, and repetitive visualization of targeted biomarkers and monitoring of related biological processes and disease progression, has recently attracted much attention of researchers and clinicians in various fields.^[^ ^1^ ^]^ The main modalities of molecular imaging could be categorized into two groups.^[^ ^2^ ^]^ Optical imaging, positron emission tomography (PET), and single photon emission CT (SPECT) are generally used for providing functional or molecular information.^[^ ^3^, ^4^, ^5^ ^]^ Meanwhile, magnetic resonance imaging (MRI), computed tomography (CT), and ultrasound (US) are commonly used for anatomic imaging.^[^ ^5^, ^6^ ^]^ The main advantage of molecular imaging is its ability to characterize and visualize diseases‐related biomarkers, and promote the use of molecular features to customize effective personalized therapies. Furthermore, owing to the non‐invasiveness of molecular imaging, these processes can be monitored in real‐time manner. The evolving of molecular imaging promises improvements in the specific and quantitative diagnosis without invasive biopsies or surgical procedures.

The two‐ or three‐dimensional result revealed by molecular imaging is usually detected and analyzed with the assistance of designed molecular probes.^[^ ^7^, ^8^ ^]^ Although molecular imaging has exhibited great potential in the diagnosis of various diseases and prediction of their prognosis, these imaging modes have some inherent limitations, including poor sensitivity (for MRI), low spatial resolution (for PET), and limited penetration depth (for fluorescence imaging).^[^ ^9^, ^10^ ^]^ However, these issues can be alleviated by using specific probes such as paramagnetic probes (for increased signal strength), iodinated contrast agents (for improved imaging contrast and resolution), and near‐infrared‐based probes (for deeper penetration depth).^[^ ^11^, ^12^ ^]^ Furthermore, taking advantage of endogenous properties of different diseases, such as pH, enzymes, and redox agents and external stimuli such as temperature and light, condition‐dependent imaging probes are designed to enable the delivering of probes to be more efficient and specific to the target site compared with normal probes. For example, the sensitivity of simple MRI contrast agents is usually low and a millimolar range of concentration of contrast agent is typically required.^[^ ^13^ ^]^ The stimuli‐activated probes could selectively accumulate at imaging sites through enzymatic amplification, thereby overcoming the obstacle of low sensitivity in magnetic resonance imaging.^[^ ^14^ ^]^ Therefore, the use of activatable imaging probes with high sensitivity are crucial to address these obstacles in molecular imaging.

Activatable imaging probes are generally consisted of three parts: targeting moiety that could specifically recognize and bind to the biomarkers, a reporter (such as paramagnetic substances, radioactive nuclides, and fluorophores) that is visible in different imaging modalities, and a linker or carrier that used for connecting the ligand with the reporter chemically. The linker used in a molecular imaging probe can couple the targeting moiety with the signal agent, which has a profound impact on the characteristics of the imaging probe.^[^ ^15^ ^]^ Stimuli‐responsive linkers have been applied to design activatable probes in molecular imaging. To date, many researchers have reported breakthrough results in molecular imaging using a range of responsive linkers to promote the potency of probes. These stimulus‐cleavable linkers can be categorized into enzyme‐responsive linkers, redox‐activation linkers, ROS‐cleavable linkers, and pH‐induced linkers.^[^ ^16^, ^17^ ^]^ Specifically, in the particular sites of pathology, such as cancer and inflammation, the dysregulation of certain enzymes,^[^ ^18^ ^]^ elevated pH levels,^[^ ^19^ ^]^ as well as differences in GSH expression are pathological features of a wide range of diseases.^[^ ^20^ ^]^ In order to achieve an ideal imaging result, the specific changes produced in these lesion regions provide the impetus for the development of related responsive cleavable linkers. The activatable probes based on these responsive linkers are designed to change the signal of the reporter from a turned ‘‘off’’ to an ‘‘on’’ state by biochemical reactions, for example, transitioning from a quenched non luminescent state to an activated luminescent state, transitioning from emission at one wavelength to emission at another wavelength, or transitioning from free diffusion to self‐assembly.^[^ ^8^ ^]^ Alternatively, these probes are also used to selectively accumulate at the imaging site by responsive cleavage of the linkers to provide sufficient signal and contrast for effective imaging.^[^ ^21^ ^]^

**SCHEME 1:**
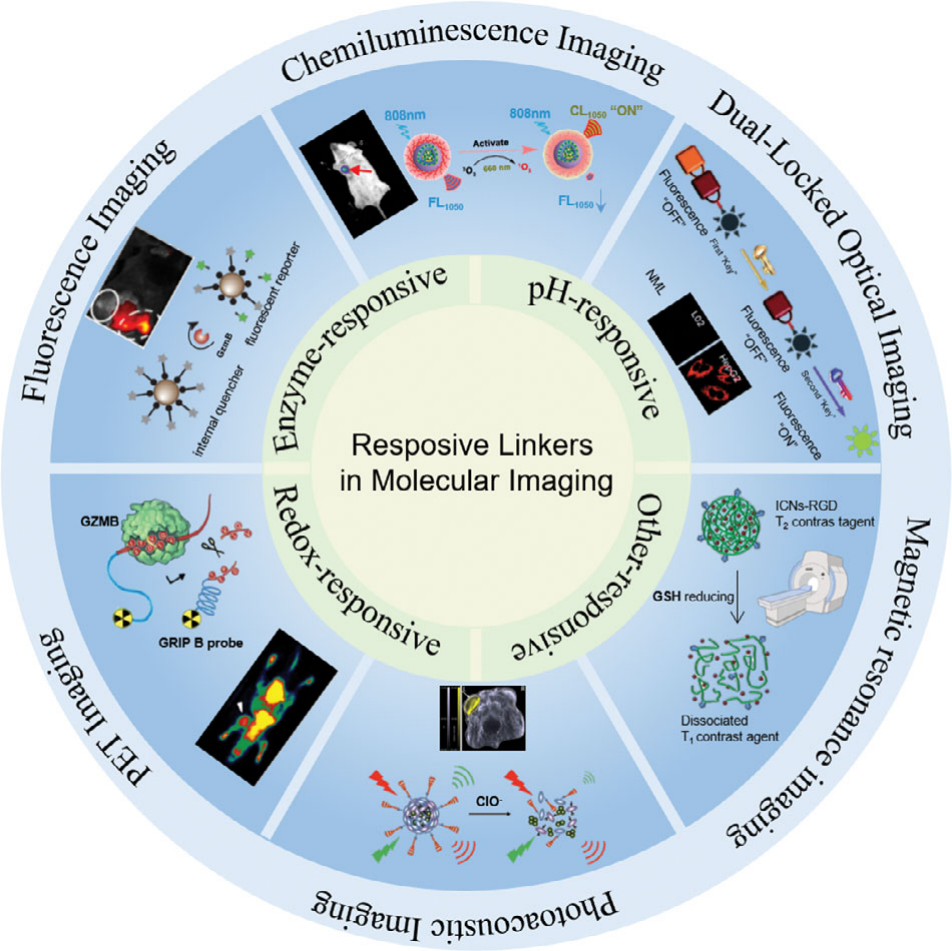
Schematic illustration of responsive linkers in molecular imaging. Category of image‐related responsive linkers (inner ring). Responsive linkers in various imaging modalities for diseases (outer ring). Reproduced with permission.^[^ ^23^ ^]^ Copyright 2019, Wiley‐VCH Verlag GmbH & Co. KGaA; Reproduced with permission.^[^ ^24^ ^]^ Copyright 2019, Wiley‐VCH Verlag GmbH & Co. KGaA; Reproduced with permission.^[^ ^25^ ^]^ Copyright 2020, American Chemical Society; Reproduced with permission.^[^ ^26^ ^]^ Copyright 2020, American Chemical Society; Reproduced with permission.^[^ ^27^ ^]^ Copyright 2020, American Chemical Society; Reproduced with permission.^[^ ^28^ ^]^ Copyright 2021, American Chemical Society; Reproduced with permission.^[^ ^29^ ^]^ Copyright 2021, American Chemical Society; Reproduced with permission.^[^ ^30^ ^]^ Copyright 2017, American Chemical Society; Reproduced with permission.^[^ ^31^ ^]^ Copyright 2019, Royal Society of Chemistry.

Most of the available reviews address the use of responsive linkers for controlled drug delivery,^[^ ^16^, ^22^ ^]^ antibody‐drug conjugates,^[^ ^17^ ^]^ tumor diagnostics and targeted therapies,^[^ ^9^ ^]^ little has been reported on the use of responsive linkers for molecular imaging. Herein, we systematically introduced recent examples and progress toward the development of stimuli‐responsive linkers for molecular imaging from a chemistry perspective in this review. Firstly, we summarize the category of responsive linkers used in molecular imaging. Subsequently, the design strategies and applications of responsive linkers in different imaging modalities are emphasized (Scheme 1). Further, we provide an outlook on the use of responsive linkers in imaging and we believe that responsive linkers hold great opportunities in molecular imaging.

## 2 CLEAVABLE LINKERS

Linkers which are able to respond to endogenic or exogenous stimuli, are commonly used in drug delivery to release preloaded therapeutics in a precision manner with a high efficacy and reduced cytotoxicity, as detailed in the reviews by Bargh et al.^[^ ^17^ ^]^ and Xue et al.^[^ ^16^ ^]^ As for molecular imaging, the responsive linkers have also shown great advantages in the imaging probes design with the capability to reveal biological activities at a molecular level, demonstrating outstanding specificity and sensitivity. Therefore, this section will give a brief introduction of the responsive linkers used in molecular imaging (e.g. fluorescence imaging (FI), chemiluminescence imaging (CLI), dual‐locked optical imaging, magnetic resonance imaging (MRI), photoacoustic imaging (PAI), and positron emission tomography (PET) imaging), of which enzyme, redox and pH responsive linkers are the most representative linkers that need to be highlighted.

### 2.1 Enzyme‐responsive linkers

Abnormal expression of specific enzymes, such as cathepsin‐B, β‐galactosidase, caspase, matrix metalloproteinase, furin and alkaline phosphatase, are all related to tumor progression and metastasis, which provide favorable potential biological targets to develop enzyme‐responsive linkers. In addition, changes of tumor microenvironment induced by cancer therapy can also be utilized for responsive imaging probe design to reveal the therapy outcome. For instance, the activation of cytotoxic T cells (CTLs) and natural killer (NK) cells during immunotherapy would lead to the secrete of excess granzyme B, which can serve as a predictive biomarker to design granzyme B‐responsive imaging probes to monitor immune activation process.^[^ ^32^ ^]^ By exploiting these enzyme‐responsive linkers, a variety of enzyme activatable molecular imaging probes have been conceived. The enzymes functionalize as triggers to specifically cleave the linkers, generating signal changes to reveal enzyme activity correspondingly, and thus facilitating biomarkers‐related diseases diagnosis, tumor therapeutical effect detection and imaging contrast improvement.

#### 2.1.1 Cathepsin‐B responsive linkers

Cathepsin is a lysosomal cysteine protease that plays a prominent role in degrading intra‐ and extracellular proteins. Cathepsin‐B is often overexpressed in malignant tumors, and involved in regulating the tumor microenvironment in multiple cancer types.^[^ ^33^ ^]^ Imaging cathepsin‐B activity is achieved by utilizing cathepsin‐B‐responsive linkers with different imaging modalities.

Gly‐Phe‐Leu‐Gly (GFLG) is a common cathepsin‐B‐responsive linker that has been effectively used in the design of fluorescence imaging and magnetic resonance imaging probes.^[^ ^16^, ^17^ ^]^ The overexpressed cathepsin‐B in the tumor microenvironment can cleave the GFLG linker between Gly and the payloads, releasing the fluorescent moieties or chemotherapy drugs in their active form. Similarly, the Phe‐Arg‐Arg‐Gly (FRRG) linker has also been employed to release cargo upon cathepsin cleavage.^[^ ^34^, ^35^ ^]^ Additionally, a Cbz‐Lys‐Lys‐p‐aminobenzyl alcohol (PABA) linker, which can be cleaved between the lys and PABA, has also been reported for fluorescence imaging of cathepsin‐B activity.^[^ ^36^, ^37^, ^38^ ^]^

Another big category of cathepsin‐B‐responsive linker is the Val‐Cit (VC) dipeptide, which has been applied to chemiluminescence imaging,^[^ ^39^ ^]^ magnetic resonance imaging,^[^ ^40^ ^]^ and photoacoustic imaging.^[^ ^41^ ^]^ The cleavage of Val‐Cit linker at Cit site results in the production of activated chemiluminophore accompanied by remarkable chemiluminescent signal,^[^ ^16^ ^]^ or self‐assembly of the remaining payload into nanoparticles with increased MR contrast or photoacoustic signals,^[^ ^34^, ^35^ ^]^ providing enhanced contrast at target sites or tissues relative to normal tissues.

#### 2.1.2 β‐Galactosidase responsive linkers

The up‐regulation of β‐galactosidase (β‐gal), a lysosomal hydrolytic enzyme, is frequently related with the onset of primary ovarian cancer and cellular senescence.^[^ ^42^ ^]^ As a result, detecting β‐gal activity is extremely important in cancer diagnosis.

β‐galactose is the most representative β‐galactosidase‐responsive linker that has been successfully designed for fluorescence imaging,^[^ ^43^ ^]^ chemiluminescence imaging,^[^ ^16^, ^41^ ^]^ and magnetic resonance imaging probes.^[^ ^42^, ^43^, ^44^ ^]^ Upon cleavage by β‐galactosidase, the fluorescent chromophore or chemiluminophore conjugated to the linker will get exposed, resulting the activation of the fluorescence or chemiluminescence signal. The cleavage of β‐galactose will also lead to the exposure of Gd‐DOTA agent to H_2_O molecules, altering the *q* value and improving the relaxivity of MR contrast agent.^[^ ^45^ ^]^ The activation or contrast enhancement of molecular imaging probes can facilitate real‐timely and sensitively detection of β‐gal activity.

#### 2.1.3 Caspase responsive linkers

Apoptosis is necessary to maintain normal cellular homeostasis, but loss control of the apoptosis process is closely related to the onset of a variety of diseases.^[^ ^46^ ^]^ Caspase‐3/7 is an important molecular target for apoptosis imaging because of its crucial role in early apoptosis.^[^ ^47^ ^]^

A specific linker known as DEVD‐X (Asp‐Glu‐Val‐Asp‐X, where X is any amino acid) can be cleaved by caspase‐3/7 at the site between X and D.^[^ ^48^ ^]^ Taking advantage of caspase‐3/7 responsive DEVD linker, activatable FI,^[^ ^49^, ^50^, ^51^ ^]^ MRI,^[^ ^51^, ^52^ ^]^ PAI,^[^ ^53^, ^54^ ^]^ and PET^[^ ^55^, ^56^ ^]^ probes have been developed to detect caspase‐3/7 activity and image apoptosis as well as evaluate therapeutical effectiveness. Generally, the cleavage of DEVD linker by caspase‐3/7 will cause separation of the fluorophore from the quencher or aggregation of remaining AIEgens to turn on fluorescence signal, or lead to spontaneous aggregation of the remaining imaging agents at target sites to generate MR/ PA/ PET signal. The process of imaging probes activation is able to improve accuracy of caspase‐3/7 activity imaging.

#### 2.1.4 Matrix metalloprotease (MMP) responsive linkers

As a member of the matrix metalloproteinases (MMPs) family, matrix metalloproteinase‐2 (MMP‐2) or matrix metalloproteinase‐9 (MMP‐9), is overexpressed in a variety of tumors and associated with tumor development, invasion, and metastasis.^[^ ^57^ ^]^ They are significant tumor biomarkers and activatable imaging probes are developed accordingly for tumor progression diagnosis.

The MMP‐2‐responsive PLGLAG or GPLGVRG linker has been utilized to construct fluorescence imaging probe for detecting MMP‐2 activity.^[^ ^54^, ^58^ ^]^ The overall mechanism by which MMP‐2 catalyzes the hydrolysis of the peptide involves four key reaction steps: (i) nucleophilic attack, (ii) hydrogen‐bond rearrangement, (iii) proton transfer, and (iv) C─N bond cleavage.^[^ ^59^ ^]^ The PLG unit in the linker is the MMP‐2 activatable site. Similarly, fluorescence signal can be restored when the linker is cleaved to aggregate the AIEgen residues or detach the quencher, reflecting the MMP‐2 activity. In addition, an MMP‐9‐responsive PRQITA linker and an MMP‐2/9‐responsive Pro‐Leu‐Gly‐Met‐Trp‐Ser‐Arg‐OH (PLGMWSR‐OH) linker have been reported to functionalize magnetic resonance probes.^[^ ^53^, ^59^ ^]^ When the linker is cleaved by MMP‐2/9, the contrast agent self‐aggregates with an increased MR signal, indicating MMP‐2/9 activity.

#### 2.1.5 Furin responsive linkers

Furin protease is a proprotein invertase located in a trans‐Golgi network that cleaves a precursor protein in a specific sequence to produce a biologically active mature protein. Furin proteases have been found to be overexpressed in head and neck squamous cell carcinoma, breast cancer, and other tumors.^[^ ^60^ ^]^ Therefore, detecting furin activity help to monitor the occurrence and progression of tumors. A furin responsive Arg‐Val‐Arg‐Arg linker (RVRR) linker has been widely used to design FI,^[^ ^61^ ^]^ MR,^[^ ^58^, ^59^ ^]^ and PET^[^ ^60^, ^62^ ^]^ imaging probes. For instance, a PyTPA AIEgen was modified with RVRR linker and the cleavage by furin from the terminal Arg would lead to self‐aggregate and AIE signal to report furin activity.^[^ ^60^ ^]^ A similar TFMB‐Arg‐Val‐Arg‐Arg‐Arg (TFMB‐RVRR) linker has also been reported to detect furin activity with *T*_2_ weighted MR scanning.^[^ ^63^ ^]^

#### 2.1.6 Alkaline phosphatase (ALP) responsive linkers

Alkaline phosphatase (ALP) is a type of secreted phosphatase that is involved in numerous critical physiological and pathological processes. Abnormal alkaline phosphatase level is related to a number of diseases, including diabetes, heart disease, bone disease, breast cancer, and prostate cancer. Therefore, real‐time monitoring of alkaline phosphatase activity provide an effective tool to detect these diseases.^[^ ^64^ ^]^ Phosphate linker is one of the most common ALP‐responsive linkers and has been widely used in the study of FI,^[^ ^65^ ^]^ MRI,^[^ ^66^ ^]^ and PAI^[^ ^61^, ^67^, ^68^ ^]^ probes. It can be cleaved in the presence of ALP to produce fluorescence, MR, or photoacoustic signals to precisely monitor ALP activity in real‐time. Additionally, a Nap‐FFFYp linker has also been reported, the cleavage of which would result in aggregation of the MR probe to produce an amplified MR signal.^[^ ^66^ ^]^

#### 2.1.7 Granzyme B responsive linkers

In addition to tumor‐associated biomarkers, granzyme B is an essential serine protease involved in cytotoxic T cells killing. Granzyme B participates in multiple anti‐tumor immune pathways which is an appealing immune‐related biomarker. Monitoring granzyme B activity not only aids in the identification of active cytotoxic T cells but also directly shows the kinetics of immune responses,^[^ ^69^ ^]^ which can be achieved by utilizing granzyme B‐responsive linkers.

Granzyme B‐responsive Ile‐Glu‐Pro‐Asp (IEPD) linker and Ile‐Glu‐Phe‐Asp (IEFD) linker have been used to couple a fluorescent molecule and a quencher, or to an inactive fluorescent molecule. The linker can be precisely cleaved at the Asp sites, restoring quenched fluorescence, or resulting in active fluorescent molecular structure transformation, with the ability to monitor the granzyme B activity and assessment of immunotherapy response in real‐time.^[^ ^32^, ^66^, ^69^, ^70^ ^]^ Furthermore, based on the tetrapeptide IEPD linker, various hexapeptides linkers such as IEPDAG, IEPDSG, IEPDSL, IEPDWL, IEPDAL, IEPDWR, and IEPDRL have been investigated with higher sensitivity to granzyme B.^[^ ^71^ ^]^ The IEPD linker is also exploited to fabricate chemiluminescence imaging probes to image NK cell activity.^[^ ^72^ ^]^ Furthermore, with the modification of VSVQ sequence, the IEPDVSVQ linker showed an improved sensitivity to granzyme B, and has been used to construct activatable PET probe.^[^ ^28^ ^]^

#### 2.1.8 Other enzyme responsive linkers

Apart from those mentioned above, other responsive linkers have been developed to design activatable molecular imaging probes, such as carbapenemase‐responsive carbapenem linker,^[^ ^73^ ^]^ fibroblast activation protein‐alpha (FAPα) ‐responsive dipeptide linker (glycine‐proline),^[^ ^74^ ^]^ γ‐glutamyl transpeptidase (GGT)‐responsive linker (γ‐Glu),^[^ ^75^ ^]^ leucine aminopeptidase (LAP)‐responsive linker (l‐leucine),^[^ ^76^ ^]^ neuraminidase‐responsive linker (*N*‐acetylneuraminic acid),^[^ ^77^ ^]^ quinone oxidoreductase‐1 (NQO1)‐responsive linker (trimethyl‐locked quinone),^[^ ^78^ ^]^ and so on. In a similar manner, once the linkers are cleaved by corresponding enzymes, the molecular structure or the aggregation state of the imaging probes will change, which will generate imaging signals at the same time.

The representative enzyme‐responsive linkers and their application in activatable imaging probes have been summarized in Table 1.

**TABLE 1:** Summary of enzyme‐responsive linkers and their usages in different activatable imaging probes.

Responsive linkers	Cargo	Responsive enzymes	Imaging modality	Cell lines/tumors	Imaging effect	Ref
GFLG	Silicon phthalocyanine (Pc158)	Cathepsin B	FI	PC3pip /PC3flu	–	[36]
VC	Adamantylidene−dioxetane	Cathepsin B	CLI	CT26/3T3	–	[39]
	Gadolinium (Gd)	Cathepsin B	MRI (*T*_2_)	MDA‐MB‐231	*T*_2_ relaxation time shorten from 361.2 to 217.2 ms	[40]
	2‐cyano‐6‐aminobenzothiazole (CBT)	Cathepsin B	PAI	MDA‐MB‐231	A 4.9‐fold or 4.7‐fold PA signal enhancement	[41]
CGKRK ‐maleimide‐VC	Adamantylidene−dioxetane	Cathepsin B	CLI	CT26/3T3	–	[39]
PABA	Fluorophore amino luciferin	Cathepsin B	FI	HeLa/MDA‐MB‐231/U87	≈73‐fold enhancement in fluorescence	[79]
β‐galactose	DCM‐NH_2_	β‐galactosidase	FI	293T/SKOV3	A low detection limit of 1.26×10^−3^ U mL^−1^	[43]
	Phenoxy‐dioxetane luminophores	β‐galactosidase	CLI	HEK293‐LacZ/HEK293‐wt	–	[80]
	Gd(III)	β‐galactosidase	MRI (*T*_1_)	GM1	A 9.5% and 45.2% increase in MR signal	[81]
	Gd(III)	β‐galactosidase	MRI (*T*_1_)	–	*q* value increased from 0 to 1 and a Δ*r*_1_ = 106% versus Δ*r*_1_ ≈ 20%	[82]
DEVD	Alexa Fluor 647 and QSY 21	Caspase 3/7	FI	HeLa/retinal ganglion cells (RGCs)	–	[49]
	Gd(III)	Caspase 3/7	MRI (*T*_1_)	HeLa	*T*_1_ relaxation time decreased from 1744.2 to 1468.0 ms	[51]
	SPIO nanoparticles	Caspase 3/7	MRI (*T*_2_)	HepG2	≈65.2% enhancement of *r*_2_ values	[52]
	Nanogapped gold nanoparticles (AuNNPs)	Caspase 3	PAI	HepG2	PA amplitude at 1250 nm gradually increased	[53]
	ICG	Caspase 3	PAI	U87MG	ΔPA ≈553 for treated tumor vs. ΔPA ≈127 for control	[54]
	[^18^F]AMBF3	Caspase 3	PET	HeLa	2.2 folds of retained [^18^F] in tumor	[56]
IETD	Tetraphenylsilole (TPS)	Caspase8	FI	HeLa	–	[50]
PLGLAG	PyTPA	MMP‐2	FI	HFL/HeLa/SKOV‐3	–	[83]
GPLGVRG	Cy5.5 and QSY21	MMP‐2	FI	MKN45/GES‐1	≈185‐fold NIR fluorescence turn‐on ratio	[58]
PRQITA	Very small iron oxide particles (VSOP)	MMP‐9	MRI (*T*_2_)	–	–	[62]
PLGMWSR‐OH	Iron oxide nanoparticles (IONPs)	MMP2/9	MRI (*T*_2_)	U87.CD4.CXCR4	160% of *T*_2_ signal enhancement	[57]
RVRR	Nitrobenzoxadiazole (NBD)	Furin	FI	MDA‐MB‐231	–	[61]
	SPIO	Furin	MRI (*T*_2_)	MDA‐MB‐468 /HEK 293T	≈81.9% increase of *r*_2_ values	[84]
	SPIO	Furin	MRI (*T*_2_)	MDA‐MB‐468	≈63.96% increase of *r*_2_ values	[63]
	CBT‐^68^Ga	Furin	PET	MDA‐MB‐468	–	[67]
Phosphate	Rhodol	ALP	FI	HeLa	–	[65]
	LET‐CyOH	ALP	PAI	HeLa	–	[64]
Nap‐FFFYp	Gd(III)	ALP	MRI (*T*_2_)	HeLa	33.9% enhancement of the *r*_2_	[66]
IEPD	5‐FAM and QSY‐7	Granzyme B	FI	B16/F10	–	[32]
	BODIPY‐FL and Dabcyl	Granzyme B	FI	Wild‐type SCC and SCC FAK (−/−)	–	[71]
	Phenoxydioxetane	Granzyme B	CLI	NK‐92/MDA‐MB‐231	–	[72]
IEFD	CyOHP	Granzyme B	FI	4T1	–	[85]
IEPDVSVQ	^64^Cu‐labeled GRIP B	Granzyme B	PET	MC38/CT26	–	[28]
Carbapenem	Dioxetane luminophore	carbapenemase	CLI	IMP‐2‐/KPC‐2‐expressing bacteria cells	–	[73]
Glycine‐proline	Adamantylidene‐dioxetane	fibroblast activation protein‐alpha (FAPα)	CLI	HepG2	Improved detection limit (0.785 ng mL^−1^)	[74]
γ‐Glu	Phenoxy‐dioxetane	γ‐glutamyl transpeptidase (GGT)	CLI	OVCAR5/U87MG	Over 800‐fold turn‐on ratio	[75]
l‐leucine	An acryl‐substituted phenoxy 1,2‐dioxetane luminophore (Int 4‐1)	Leucine aminopeptidase (LAP)	CLI	LO2/HepG2 cells	–	[76]
*N*‐acetylneuraminic acid	Phenoxy‐dioxetane	neuraminidase	CLI	MDCK	–	[77]
Trimethyl‐locked quinone	Phenoxy‐dioxetane	Quinone oxidoreductase‐1 (NQO1)	CLI	A549/H596	–	[78]

Abbreviations: CBT, 6‐amino‐2‐cyanobenzothiazole; Gd, gadolinium; IONPs, iron oxide nanoparticles; NBD, nitrobenzoxadiazole; TPS, tetraphenylsilole; VSOP, very small iron oxide particles.

### 2.2 Redox‐responsive linkers

Maintaining the dynamic balance of redox environments is critical for normal organism metabolism, as redox balance is regulated by biological oxidants and reluctance. The disorder of redox environments is associated with the occurrence and progression of cancer, diabetes, inflammation, and other diseases.^[^ ^86^ ^]^ The primary biological redox biomarkers that are involved in regulating organisms’ redox status and numerous physiological processes include reactive oxygen species (ROS), reactive nitrogen species (RNS), and reactive sulfur species (RSS). Imaging and monitoring of redox biomarkers are critical for disease diagnosis and treatment,^[^ ^87^ ^]^ which can be achieved by using redox‐responsive linkers, such as ROS, RNS, and RSS‐responsive linkers, to construct imaging probes.

#### 2.2.1 Reactive oxygen species (ROS) responsive linkers

Reactive oxygen species (ROS) are chemical species that are formed as a result of incomplete oxygen reduction. It mainly includes singlet oxygen (^1^O_2_), hydrogen peroxide (H_2_O_2_), superoxide anion (O^2•−^), and hypochlorite (ClO^−^). These chemical species play vital role in regulating the various physiological functions of living organisms.^[^ ^88^ ^]^ Imaging probes functionalized with ROS‐responsive linkers are able to track ROS in real‐time.

Currently, ^1^O_2_‐responsive adamantine‐1,2‐dioxetane linker,^[^ ^25^ ^]^ and adamantine‐enol‐ester linker^[^ ^89^ ^]^ have been utilized for chemiluminescence imaging of ^1^O_2_ activity. When reacting with ^1^O_2_, the two linkers will be oxidized to phenol‐dioxetane species, which can spontaneously decompose into an electronically excited benzoate ester, accompanied by the production of strong chemiluminescence signals for detecting ^1^O_2_.

Apart from ^1^O_2_, H_2_O_2_, and O^2•−^‐responsive linkers have also been extensively applied for H_2_O_2_ and O^2•−^ detection. Among them, luminol linker is typically employed in the design of chemiluminescence probes,^[^ ^89^, ^90^, ^91^ ^]^ which can be oxidized to the excited state of 3‐aminophthalate dianion in the presence of H_2_O_2_.^[^ ^92^ ^]^ In addition, aryl boronate linker^[^ ^92^, ^93^, ^94^, ^95^ ^]^ and benzeneboronic acid pinacol ester linker^[^ ^96^ ^]^ have also been developed for H_2_O_2_ imaging. For O^2•−^ detecting, a O^2•−^‐responsive imidazopyrazinone linker has been successfully applied to chemiluminescence probes to generate signals upon oxidation of imidazopyrazinone to dioxane.^[^ ^97^ ^]^

ROS‐responsive thioketal (TK) linkers^[^ ^98^ ^]^ and ClO^−^‐responsive ITTC linkers^[^ ^99^ ^]^ have also been reported to image ROS, upon cleavage and release of active fluorescent molecules.

#### 2.2.2 Reactive nitrogen species (RNS) responsive linkers

Reactive nitrogen species (RNS), mainly including ONOO^−^ and NO, play an important role in the progression of cancer or other diseases.^[^ ^100^ ^]^ In situ imaging of ONOO^−^ and NO is of great significance for studying the critical roles of ONOO^−^ and NO in disease progression. To achieve this, an ONOO^−^‐responsive A1094 linker and a bulky borane linker have been reported to design activatable FI^[^ ^101^ ^]^ and PA^[^ ^102^ ^]^ probes, respectively. NO imaging can be achieved by using an o‐aminophenol linker as demonstrated in activatable PA probes that *N*‐nitrosation of the linker would lead to a wavelength shift.^[^ ^98^, ^99^ ^]^

#### 2.2.3 Reactive sulfur species (RSS) responsive linkers

Reactive sulfur species (RSS) mainly include H_2_S and GSH. H_2_S is regarded as a biological reaction regulator that controls the redox state and a number of physiological processes in living organisms.^[^ ^103^ ^]^ Reactive PA probes have been developed to imaging H_2_S activity using H_2_S‐responsive linkers, such as benzoic ester and AzHD linker.^[^ ^104^, ^105^ ^]^ GSH is another common endogenous antioxidant and GSH deficiency can result in a range of disorders (e.g. aging, cardiovascular disease, cancer, and so on).^[^ ^106^ ^]^ The most frequently used GSH‐responsive linker is disulfide, which have been utilized to build FI,^[^ ^107^ ^]^ MR,^[^ ^108^, ^109^ ^]^ and PA^[^ ^26^ ^]^ probes to precisely image GSH.

The discussed redox‐responsive linkers and their application in activatable imaging probes have been summarized in Table 2.

**TABLE 2:** Summary of redox‐responsive linkers and their application in activatable imaging probes.

Responsive linkers	Cargo	Responsive redox conditions	Imaging modality	Cell lines/tumors	Imaging effect	Ref
Adamantine‐1,2‐dioxetane	Phenoxy‐dioxetane and dicyanomethylchromone	^1^O_2_	CLI	HeLa/MCF‐7/4T1	A 1011‐fold turn‐on of chemiluminescent signal	[25]
Adamantine‐enol‐ester	Dioxetane luminophores	^1^O_2_	CLI	HeLa	–	[89]
Luminol	AuNPs@g‐C_3_N_4_	H_2_O_2_	CLI	SKOV3ip/HMrSV5	–	[90]
Aryl boronate	Dioxetane luminophores	H_2_O_2_	CLI	HEK293‐Lac‐Z	–	[93]
	Heptamethine carbocyanine	H_2_O_2_	FI, PAI	MDA‐MB‐231/MCF7	–	[94]
	Xanthenone	H_2_O_2_	FI	HEK	–	[110]
Benzeneboronic acid pinacol ester	Aza‐BODIPY	H_2_O_2_	PAI	A549	The PA_825_/PA_725_ value increased to 1.49 ± 0.021, ≈5 fold higher	[96]
Imidazopyrazinone	Tetraphenylethene (TPE)	O^2•−^	CLI	HL‐7702	–	[111]
Thioketal (TK)	Photosensitizer HPPH	H_2_O_2_	FI	CT26	–	[98]
ITTC	PDF	ClO^−^	FI	3T3	–	[99]
A1094	Ag_2_S quantum dots (QDs)	ONOO^−^	FI	Human umbilical vein endothelial cells	–	[101]
Bulky borane	Boronate‐caged boron‐dipyrromethene dye (BBD)	ONOO^−^	PAI	HeLa/4T1	–	[102]
o‐aminophenol	Aza‐BODIPY dye	NO	PAI	LPS‐mediated inflammation model	A 1.9‐fold increase at 680 nm and a 1.3‐ fold ratiometric turn‐on	[112]
Benzoic ester	Meso‐hydroxyltricarbo‐heptamethine cyanine	H_2_S	PAI	–	–	[104]
AzHD	NIR dye AzHD	H_2_S	PAI	4T1/HCT116	–	[105]
Disulfide	BTMP	GSH	FI	HepG2/MCF‐7/HeLa	–	[107]
	Gd(III)	GSH	MRI (*T*_1_)	PC3pip and PC3flu	53.0% increase in *r*_1_ values	[108]
	ESIONPs	GSH	MRI (*T*_2_)	U87‐MG	*r*_1_ value increased from 5.56 to 7.40 mm ^−1^ s^−1^, *r*_2_ value decreased from 103.01 to 14.36 mm ^−1^ s^−1^	[109]
	IR806	GSH	PAI	HeLa		[26]

Abbreviations: BBD, boronate‐caged boron‐dipyrromethene dye; QDs, quantum dots; TPE, tetraphenylethene.

### 2.3 pH‐responsive linkers

Abnormal extracellular pH is associated with a variety of pathological states such as tumor progression, ischaemic stroke, infection, inflammation and so on.^[^ ^113^ ^]^ Acidic extracellular pH (pH 6.2–6.9) is considered to be one of the significant indications of malignancy due to the enhanced glycolysis and poor perfusion, which create an acidic extracellular environment that promotes tumor growth, invasion, and metastasis.^[^ ^114^ ^]^ Therefore, accurate real‐time monitoring of the dynamic changes of extracellular pH can provide important information not only for the study of pathological processes, but also for the theranostics of cancer and other diseases. Various pH‐responsive linkers such as 3,9‐bis(3‐aminopropyl)−2,4,8,10‐tetraoxaspiro[5.5]undecane(ATU) linker,^[^ ^115^ ^]^ piperazine linker,^[^ ^116^ ^]^ phenol,^[^ ^117^ ^]^ PDPA,^[^ ^30^ ^]^ β‐thiopropionate,^[^ ^118^ ^]^ mPEG‐*b*‐P(DPA‐DE) LG,^[^ ^119^ ^]^ glycol chitosan (GC) linker^[^ ^24^ ^]^ etc. have been utilized in the design and development of pH‐responsive imaging probes for monitoring pH changes, accurately imaging tumors, detecting diseases, and monitoring drug release.

The representative pH‐responsive linkers and their application in activatable imaging probes have been summarized in Table 3.

**TABLE 3:** Summary of pH‐responsive linkers and their application in activatable imaging probes.

pH‐responsive linkers	Cargo	Imaging modality	Cell lines/tumors	Imaging effect	Ref
3,9‐bis (3‐aminopropyl) ‐2,4,8,10‐tetraoxaspiro[5.5] undecane(ATU) linker	Cy5 and quencher BHQ2	FI	A549	–	[115]
Piperazine	Perylenediimides (PDIs)	FI	4T1	–	[116]
Pohenol	Aza‐BODIPY	FI	BV2/PC12 cells middle cerebral artery occlusion /reperfusion (MCAO/R) rats	–	[117]
PDPA	Rapamycin, Ce6, Gd^3+^	FI	PC12 cells/tMCAO rats	–	[30]
β‐thiopropionate	ES‐MIONs	MRI (*T*_1_)	U‐87 MG	*r*_1_ value was up to 8.80 mm ^−1^ s^−1^ at 1.5 T, ΔSNR is 203.4 ± 15.1% at 12 h postinjection	[118]
mPEG‐b‐P(DPA‐DE) LG	Fe_3_O_4_	MRI (*T*_2_)	HepG2/cerebral ischemia disease rat	Enhanced negative contrast with an *r*_2_ relaxivity of ≈106.7 mm ^−1^ s^−1^	[119]
Glycol chitosan (GC)	Dextran‐stabilized SPIO nanoparticles	MRI (*T*_2_)	T6‐17	–	[24]
Amine‐substituted perdiimide (PDI) derivative	NIR dye (IR825), DOX	PAI	U87MG	The ratiometric PA signal at pH 5.0 was around 1.77‐ and 2.38‐fold higher than those at pH 6.5 and 7.4	[132]
Phenazine	dye (PIOH)	PAI	MDR‐231	–	[133]

Abbreviations: GC, glycol chitosan; PDI, perdiimide.

### 2.4 Other responsive linkers

Light is a particularly attractive exogenous stimulus because of its easily modulated intensity, non‐invasiveness, and fine temporal and spatial control.^[^ ^120^ ^]^ By using different light sources, such as ultraviolet (UV), visible, near‐infrared (NIR) light, irradiation at the target site can exhibit more controllable linker cleavage and photoactivatable fluorescence imaging.^[^ ^121^ ^]^ A number of light‐responsive linkers such as spiropyran (SP),^[^ ^122^ ^]^ 2‐nitrobenzyl,^[^ ^123^ ^]^ phenacyl,^[^ ^124^ ^]^ or 7‐methoxylcoumarin‐4‐ylboron‐dipyrromethene (BODIPY),^[^ ^125^ ^]^ PhL^[^ ^126^ ^]^ linker, have been developed for the design of light‐responsive fluorescence imaging probes, which can be used for precise imaging of tumors, monitoring drug release and cancer treatments.

In addition, detection of some important metal ions such as copper (Cu^2+^), zinc (Zn^2+^), and mercury (Hg^2+^) are also of great interest to researchers due to their diverse biological roles. Copper plays an important role in various biological processes, and copper deficiency can lead to osteoporosis, hyperthyroidism, and coronary heart disease.^[^ ^127^ ^]^ Zinc ions are important in enzyme catalysis, apoptosis and neurotransmission.^[^ ^128^ ^]^ Mercury is recognized as the most toxic metal ion to humans among the heavy metal transition metal ions. It poses a significant risk to various organs such as the kidneys, brain, nervous system, and immune system.^[^ ^129^ ^]^ Hence, there is a pressing need for a simple and rapid method to detect these ions. Fluorescence imaging offers several advantages including high sensitivity, simplicity, real‐time monitoring, and fast response time. Researchers have utilized copper, zinc, and mercury ions‐responsive linkers such as t‐butyl pyrrole,^[^ ^31^ ^]^ spironolactone,^[^ ^130^ ^]^ salicylaldimine Schiff base,^[^ ^128^ ^]^ spirolactam^[^ ^131^ ^]^ linkers to develop activatable fluorescent imaging probes for ions detection accordingly.

The representative light‐responsive and metal ions‐responsive linkers and their application in activatable imaging probes have been summarized in Table 4.

**TABLE 4:** Summary of light/ions responsive linkers and their application in activatable imaging probes.

Light/ions linkers	Cargo	Responsive conditions	Imaging modality	Cell lines/tumors	Imaging effect	Ref.
Spiropyran (SP)	Graphene oxide (GO)	UV light (365 nm)	FI	A549/MDCK	‐	[122]
	Tetraphenylethylene (TPE)	UV light (365 nm)	FI	HeLa	–	[123]
2‐nitrobenzyl, phenacyl, or 7‐methoxylcoumarin‐4‐yl	Salicylaldehyde hydrazones	UV light (365 nm)	FI	MCF‐7	–	[124]
Boron‐dipyrromethene (BODIPY)	BODIPYchlorambucil (BC) prodrug, IR783	Light (560 nm)	FI	HCT116	–	[125]
PhL	LiYF4:Yb3+/Tm3+@SiO2, FITC‐BSA	Light (980 nm)	FI	Bovine cells	–	[126]
t‐butyl pyrrole	Rhodamine	Cu^2+^	FI	MCF‐7	–	[31]
Spironolactone	Fluoran dye	Cu^2+^	FI	HeLa	–	[130]
Salicylaldimine Schiff base	PSaAEMA‐co‐PMPC	Zn^2+^	FI	HeLa	–	[128]
Spirolactam	*N*'‐(4‐methylthio)butan‐2‐ylidene) rhodamine B hydrazide	Hg^2+^	FI	HeLa	–	[131]

Abbreviations: BC, BODIPY chlorambucil; BODIPY, boron‐dipyrromethene; GO, graphene oxide; SP, spiropyran; TPE, tetraphenylethylene.

## 3 IN MOLECULAR IMAGING

### 3.1 In optical imaging

Optical imaging is a convenient tool for visualizing biological processes and disease progression and has played an important role in biomedical research and image‐guided theranostics. However, its image quality is largely limited by the low signal‐to‐background ratio (SBR) and low penetration depth caused by light scattering and tissue autofluorescence.^[^ ^87^ ^]^ To overcome this issue and further facilitate precise image‐guided theranostics, stimuli‐responsive linkers have been applied to design activatable optical imaging probes for monitoring biomarkers related immunoactivation, disease progression, drug release, and cancer treatment efficacy. These linkers can be cleaved via exposure to the specific tumor microenvironment conditions (e.g. disease‐associated enzymes, glutathione (GSH), RONS etc.) or specific light and metal ions, leading to the activation and turn‐on of the fluorescence signals. Thus, cleavage of linkers causes the followed activation of fluorescence signals restricted to local tumor lesions while remained silent in normal tissues, which resulted in higher SBR values and better accuracy. Therefore, it is of great significance to utilize responsive cleavable linkers to specifically activate optical imaging probes for highly sensitive and high‐resolution imaging. Herein, we summarize the applications of responsive linkers in optical imaging including fluorescence imaging, chemiluminescence, and dual‐locked imaging, revealing the potential for responsive linkers.

#### 3.1.1 Fluorescence imaging

Fluorescence imaging has attracted wide attention for its excellent biosafety, high sensitivity, dual spatiotemporal resolution, real‐time monitoring ability, and non‐invasive advantages.^[^ ^134^, ^135^ ^]^ However, some “always‐on” fluorescent probes produce a non‐specific signal in normal tissues, which may cause false‐positive signals and reduce detection sensitivity. Hence, responsive linkers such as enzyme‐responsive linkers, glutathione‐responsive linkers, RONS‐responsive linkers, pH‐responsive linkers, light‐responsive linkers, ions‐responsive linkers etc. can be exploited to develop activatable fluorescence imaging probes, which can achieve high specificity by turning on the fluorescence signals only under specific tumor microenvironment or pathological conditions.

**FIGURE 1:**
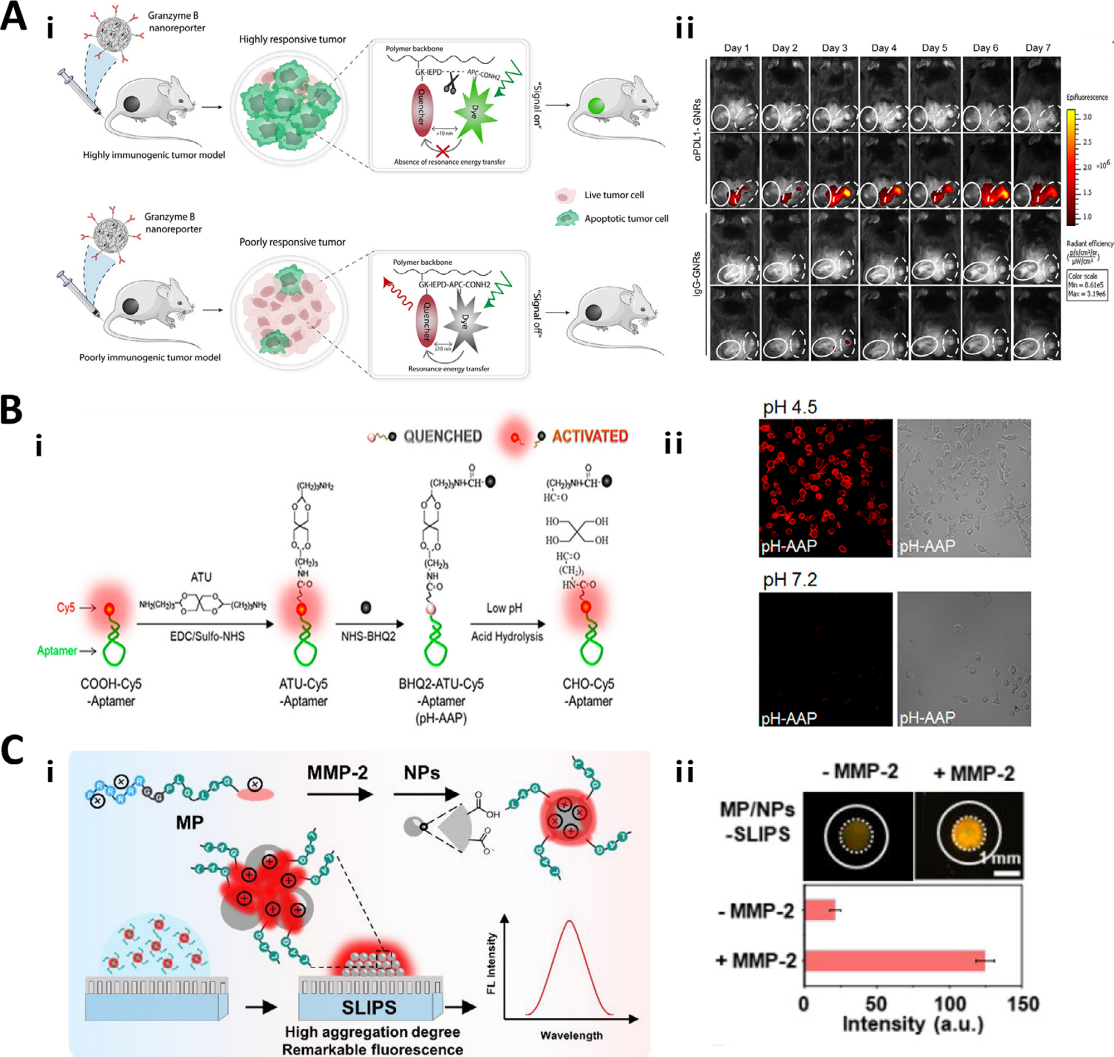
Responsive linkers for fluorescence imaging. A) Granzyme B‐responsive linker used for real‐timely monitoring of CTL activity. GrB cleaves the IEPD sequence and activates the fluorescent signal; Representative fluorescence images of MC38 and B16/F10 tumors in mice from different GNR treatment groups, showing time‐course fluorescence activation. Reproduced with permission.^[^ ^32^ ^]^ Copyright 2020, American Association for the Advancement of Science. B) pH‐responsive linker used for A549 tumor fluorescence imaging. Construction of pH‐activatable aptamer probe (pH‐AAP) and its acid‐responsive mechanism; pH‐AAP was capable of lighting up the target cells with bright red fluorescence surrounded at pH 4.5 rather than pH 7.2, due to the acid‐mediated hydrolysis of acetal groups. Reproduced with permission.^[^ ^115^ ^]^ Copyright 2019, American Chemical Society. C) MMP‐2‐responsive linker used for detecting MMP‐2. Design of modular peptide‐conjugated AIEgen MP and developing the MP/NPs‐SLIPS sensing system for sensitively detecting tumor marker MMP‐2; The fluorescent images of MP/NPs‐SLIPS with and without MMP‐2; The fluorescent images of MP/NPs‐SLIPS with MMP‐2 during its self‐assembly process. Reproduced with permission.^[^ ^83^ ^]^ Copyright 2021, American Chemical Society.

Compared to normal tissues, tumor tissues overexpress various enzymes especially proteases. Certain proteases, such as granzyme B, is an important protease involved in T cell killing.^[^ ^69^ ^]^ Some granzyme B‐responsive linker based fluorescent probes have been reported to monitor granzyme B activity in recent years. For instance, Nguyen and co‐workers used a granzyme B‐responsive IEPD linker to design a granzyme B‐responsive nanoreporter (GNR) by conjugating a dye‐quencher (5‐carboxyfluorescein‐QSY‐7) to a polymer backbone (PIMA) for tracking granzyme B activity and further monitoring the immunotherapy efficacy of MC38 colon adenocarcinoma tumor. In highly immunogenic M38 tumors, the released granzyme B cleaved the IEPD linker and led to the activation of fluorescence signal which could directly monitor granzyme B activity that correlated with T cell activity to measure the kinetics of the immune response (Figure 1A).^[^ ^32^ ^]^ In a similar way, He et al. connected the granzyme B‐responsive IEFD and IEPD linker to a NIR hemi‐cyanine dye (CyOH) containing hydrophilic polyethylene glycol (PEG) chains to develop two near‐infrared macromolecular reporters (CyGbP_F_ and CyGbP_P_). The two probes are non‐fluorescent due to the diminished electron‐donating ability of the oxygen atom in CyOH. Upon cleavage by granzyme B, the probes were converted to CyOHP, resulting in enhanced near‐infrared fluorescence signal and hence allowing in situ assessment of immunotherapy response in 4T1 tumor‐bearing mouse.^[^ ^85^ ^]^ Likewise, Mac et al. exploited the granzyme B‐responsive IEFD linker to engineer a granzyme B nanosensors for non‐invasive early detection of acute transplant rejection. In the ACR skin graft mouse model, the nanosensor accumulated in the allogeneic transplant tissue, where the IEFD linker was cleaved by granzyme B, releasing a fluorescent reporter into the recipient's urine for non‐invasive detection of anti‐graft T cells activity.^[^ ^23^ ^]^ In addition, Scott's group used a granzyme B‐responsive IEPDAL hexapeptide linker to couple the BODIPY‐FL fluorophore with ethylenediamine‐Dabcyl quencher to prepare the enzyme‐responsive fluorescent probe. In the presence of granzyme B, the linker was cleaved and the fluorescence of quenched fluorophore was recovered, permitting real‐time fluorescence monitoring of T cell‐mediated anticancer activity in mouse squamous cell carcinoma tumors and in tumors from lung cancer patients.^[^ ^71^ ^]^

Apart from granzyme B‐responsive linkers, other enzymes (e.g. cathepsin, β‐galactose, furin) responsive linkers have also been exploited to design activatable fluorescence imaging probes. For example, Luo et al. exploited a cathepsin‐responsive GLFGC linker to conjugate a silicon phthalocyanine (Pc158) to gold nanoparticles (AuNPs) which could be used for prostate tumor imaging. The AuNP‐Pc158 conjugates were completely quenched, but upon interaction with the cathepsin overexpressed in prostate tumor, the GLFGC linker was cleaved and released the free Pc158, thereby eliminating the quenching effect and switching on the fluorescent signal for prostate tumor detecting with a high specificity and sensitivity.^[^ ^36^ ^]^ Fan et al. designed a very sensitive near‐infrared probe (DCMCA‐β gal) by using the β‐glycoside linker which could be cleaved under the action of galactosidase overexpressed in ovarian cancer and release the NIR chromophore DCM‐NH_2_, producing a clear fluorescence signal for real‐time tracking of β‐gal activity in ovarian tumors.^[^ ^43^ ^]^ Li et al. decorated a furin responsive RVRR linker to a Phe‐Phe‐Phe (FFF) tripeptide sequence tailed NBD fluorophore to form a chimeric peptide probe RVRR‐FFF‐NBD (C‐3), which could self‐assemble into stable micelles for detection of furin. After being endocytosed by MDA‐MB‐231 cells, the overexpressed furin cleaved the RVRR linker, releasing the fluorescent fragment FFF‐NBD to emit green fluorescence.^[^ ^61^ ^]^

In addition to taking advantage of enzymes overexpression in tumor tissues, responsive linkers based on other endogenous stimuli (e.g. GSH, RONS, ClO^−^ etc.) have also been successfully utilized for stimuli‐responsive fluorescence imaging. For example, a glutathione‐responsive disulfide (─S─S─) linker was used by Ye et al. to design a theranostic prodrug (BTMP‐SS‐PTX) for HeLa tumor imaging. It was found that the linker could be cleaved by an intracellular high GSH concentration and subsequently release free paclitaxel (PTX) and visible 2‐(benzo[d]thiazol‐2‐yl)−4‐methoxyphenol (BTMP) with obvious fluorescence, which was good indicator for the drug uptake and therapeutic effects in HeLa tumors.^[^ ^107^ ^]^ ROS‐responsive thioketal (TK) linker was used in a study by Hao et al. to design a platinum nanozyme (PtNP)‐loaded ROS‐responsive prodrug nanoparticle (CPT‐TK‐HPPH/Pt NP). Under 660 nm laser irradiation, the TK linker was degraded due to the production of ROS, thus, releasing the HPPH with fluorogenicity, which enabled visualization of the NP uptake in CT26 cells and the tumor targeting in CT26 tumor‐bearing BALB/C mice.^[^ ^98^ ^]^ In addition, an ONOO‐ activatable A1094 linker was reported by Wang's group to design an ONOO^−^ activatable NIR‐II fluorescent nanoprobe (V&A@Ag_2_S) for detecting traumatic brain injury (TBI). Upon treatment with ONOO^−^, the A1094 linker was oxidized, turning on the fluorescence of Ag_2_S QD, providing a convenient approach for early assessment of TBI.^[^ ^101^ ^]^ Moreover, a ClO^−^ responsive linker (ITTC) was developed by Fan et al. for designing a ClO^−^ activated NIR‐II fluorescent semiconductor nanoprobe which blended the ITCC linker with semiconductor polymer. After treatment with analyte, the ITTC linker was degraded, resulting in the recovery of fluorescence of the nanoprobes, allowing imaging of ClO^−^ mediated inflammation in inflamed mouse model.^[^ ^99^ ^]^

Moreover, various pH‐responsive linkers have been explored to design fluorescence imaging probes with high sensitivity to acidic microenvironments. For example, Shi et al. employed a pH‐responsive 3, 9‐bis(3‐aminopropyl)−2, 4, 8, 10‐tetraoxaspiro[5.5]undecane(ATU) linker to connect fluorescent dye Cy5 and a quencher BHQ2 to construct a pH‐Activatable Aptamer Probe (pH‐AAP). In the acidic tumor microenvironment, cleavage of the ATU linker released active Cy5, restoring fluorescence to achieve specific A549 tumor imaging (Figure 1B).^[^ ^115^ ^]^ In another example, Li et al. modified a near‐infrared (NIR)‐absorbing perylenediimide with a pH‐responsive piperazine linker to construct pH‐responsive perylenediimide nanoparticles (PPDI‐NPs). Protonation of the piperazine linker in the acidic tumor environment blocked the photo‐induced electron transfer (PET) process of the perylene chromophore, leading to pH‐responsive NIRF emission (760 nm) and a high contrast between 4T1 tumor and normal organs.^[^ ^116^ ^]^ Beside tumor imaging, the pH‐responsive linkers can also enable ischemic stroke (IS) detection and drug release monitoring. For instance, Yao and coworkers loaded a pH‐responsive phenol group modified Aza‐BODIPY in amphipathic liposomes to prepare a pH‐responsive NIR fluorescent probe (BOD@Lip) to detect the degree of IS in vivo. The phenol group could be converted to phenolate in response to the abnormal acidic environment in ischemic tissues, leading to an increased fluorescence signal at 712 nm, which allowed visualization of the extent of IS in MCAO/R rats.^[^ ^117^ ^]^ Cheng et al. utilized a pH‐sensitive PDPA linker to prepare a pH‐sensitive amphiphilic block copolymer (mPEG‐b‐P(DPA‐co‐HEMA)‐Ce6) which could self‐assemble into nanoparticles (RAPA/Gd3+@NPs) in the presence of drug rapamycin (RAPA) and Gd^3+^. Degradation of this pH‐sensitive PDPA segment in acidic ischaemic brain tissue of the tMCAO rat model allowed the nanoparticles to dissociate and release the RPRA and Ce6, allowing drug distribution monitoring and NIR imaging of cerebral ischaemia.^[^ ^30^ ^]^

Ions‐responsive linkers have also been used to develop fluorescence imaging probes with high sensitivity to corresponding ions. For example, Guo et al. attached a copper ion (Cu^2+^)‐responsive t‐butyl pyrrole linker to a rhodamine fluorophore to construct a fluorescent probe (L1) for imaging Cu^2+^in MCF7 cells. In the presence of Cu^2+^, the t‐butyl pyrrole linker underwent a ring‐opening reaction with significantly enhanced fluorescence emission at 545 nm.^[^ ^31^ ^]^ Similarly, Qiu et al. used a Cu^2+^‐responsive spironolactone linker to design a novel Cu^2+^ fluorescent probe (FLACu) based on fluoran dye core for the detection of exogenous Cu^2+^ in HeLa cells. When exposed to Cu^2+^, the Cu^2+^ induced ring opening and hydrolysis of the spironolactone linker, switching on the fluorescent signal and allowing Cu^2+^ trace in living HeLa cells.^[^ ^130^ ^]^ Additionally, some zinc ions and mercury ions‐responsive linkers have also been reported. Yan et al. utilized zinc ion (Zn^2+^)‐responsive salicylaldimine Schiff base linker to synthesize a copolymer PSaAEMA‐co‐PMPC. Zn^2+^ can be coordinated with salicylaldimine Schiff base linker, resulting in strong fluorescence emission (450 nm) forZn^2+^ detecting in HeLa cells.^[^ ^128^ ^]^ Chen et al. used N'‐(4‐methylthio)butan‐2‐ylidene) rhodamine B hydrazide containing a mercury ion (Hg^2+^)‐responsive spirolactam linker to develop a novel chemosensor (MTRH) for in situ real‐time monitoring of Hg^2+^ in HeLa cells. In the presence of Hg^2+^, the spironolactam group opened the ring and caused a significant increase in the fluorescence signal at 580 nm.^[^ ^131^ ^]^

Besides, linkers have also been used in the design of aggregation‐induced emission luminogens (AIEgens). The linkers can be specifically cleaved upon reaction with related biomarkers, and produce hydrophobic AIE residual for aggregation, thus emitting strong fluorescence for imaging at target sites. For example, Wu et al. used an MMP‐2 responsive PLGLAG linker to connect the AIEgen PyTPA with hydrophilic peptide unit RRRRRR to form MP/NPs‐SLIPS. Under reaction with MMP‐2 in HeLa cell, linkers were cleaved to generate hydrophobic PyTPA residues, which could aggregate to emit strong fluorescence signal (Figure 1C).^[^ ^83^ ^]^ Li et al. employed a caspase3/7 responsive DEVD linker to design a caspase probe 1 (CP1) for apoptosis imaging. In response to caspase3/7, the water‐soluble DEVD linker was cleaved, and the remaining Gd(III)‐AIEgen (Gd‐AIE) conjugates aggregated, turning on the fluorescence signal which was successfully tested by fluorescence imaging of apoptotic cells.^[^ ^51^ ^]^ Zhang et al. used a β‐gal responsive glycosidic linker to synthesize an AIE fluorescent probe (TPh‐PyBz‐β‐gal) in HepG2 cells. After cleavage of glycosidic linker in the presence of β‐gal, AIE active fluorophores formed and aggregated at 606 nm, which showed selective and sensitive imaging abilities for β‐gal in living cells.^[^ ^136^ ^]^

In addition, light‐responsive linkers can also be used for the construction of responsive AIEgens based on light‐triggered linker cleavage and photoswitchable fluorescence. For example, Nahain et al. attached a light‐responsive spiropyran (SP) linker to dopamine‐modified targeting ligand hyaluronic acid (HA) to prepare functionalized graphene (rGO/HA‐SP). Under UV irradiation, the colorless SP exhibited a susceptible ring‐opening reaction, resulting in the formation of a colored merocyanine (MC) form due to the heterocleavage of the helical carbon–oxygen bond, thereby exhibiting light‐induced in vivo imaging.^[^ ^122^ ^]^ Similarly, Lin et al. attached the SP linker to AIE luminogen tetraphenylethylene (TPE) to create poly(NIPAM‐co‐TPE‐SP), which can produce AIEgens‐proportional fluorescence between green TPE and red MC emission at 517 and 627 nm upon light irradiation, allowing cellular imaging of CN^−^ in HeLa cells.^[^ ^123^ ^]^ Lu et al. employed the light‐responsive 2‐nitrobenzyl, phenylacyl, or 7‐methoxycoumarin‐4‐yl linker to cage fluorophores (salicylaldehyde hydrazone) and created three photoactivated AIE fluorophores for light‐activated MCF‐3 cell imaging. After removing the linkers with 365 nm UV irradiation, the hydroxyl and excited‐state intramolecular proton transfer (ESIPT) could be recovered to restore strong fluorescence emission in green (521 nm), yellow (541 nm), and orange (566 nm).^[^ ^124^ ^]^

Light‐triggered linker cleavage can also be exploited for monitoring drug release and cancer therapy. For example, by using the light‐responsive boron‐dipyrromethene (BODIPY) linker, Long et al. synthesized a BODIPYchlorambucil (BC) prodrug and prepared photoresponsive nanoassemblies with near‐infrared dye IR783. Under 530 nm light irradiation, the BODIPY linker degraded, resulting in the decomposition of the nanoparticles and the release of free chlorobenzene (Cb) drugs, whereas the near‐infrared fluorescence emission of IR783 in the nanoparticles exhibited a “ON‐to‐OFF” pattern, allowing the in situ detection of drug release in the HCT116 tumor‐bearing mice.^[^ ^125^ ^]^ Jalani et al. used photo‐cleavable linker (PhL) to crosslink with chitosan (CH) hydrogel and then wrapped it in LiYF4:Yb^3+^/Tm^3+^@SiO_2_ to encapsulate fluorescent‐bovine serum albumin (FITC‐BSA), obtaining UCNPs@GPS@SiO_2_@CH. Under NIR radiation (980 nm), the UCNPs upconverted the NIR light to UV light to cleave the PhL linker, leading to the dissociation of hydrogel and the instantaneous release of the encapsulated FITC‐BSA. Meanwhile, the upconverted PL emission spectra of the UCNPs have a strong NIR peak at 792 nm, thus allowing tracking drug release in bovine cells.^[^ ^126^ ^]^

#### 3.1.2 Chemiluminescence imaging

Chemiluminescence (CLI) is a luminescent phenomenon where light is generated through chemiexcitation during the chemical reaction, which is a radiative relaxation process of generated intermediates, accompanied by photon emission.^[^ ^137^ ^]^ Unlike fluorescence imaging techniques, chemiluminescence imaging provides ultra‐high sensitivity, eliminating background noise from biological tissue due to the absence of external light excitation, allowing deep tissue imaging with extremely high signal‐to‐noise ratios. In recent years, various linkers, such as ROS‐responsive linkers, enzyme‐responsive linkers and others have been used in the design of chemiluminescent imaging probes to specifically turn on the emission of quenched chemiluminophores for the detection of biomarkers in tumor cells or a certain pathological pathway.

Reactive oxygen species (ROS) are closely related to a range of diseases such as cancer, inflammation and neurodegenerative diseases etc., and play an essential role in regulating various physiological functions of living organisms.^[^ ^138^ ^]^ Imaging and monitoring ROS in vivo is of great significance for the diagnosis and treatment of clinical diseases. However, ROS is extremely reactive, making it difficult to detect ROS in real‐time. To solve this problem, in 2017, Shabat's group developed a ROS cleavable adamantine‐1,2‐dioxetane linker, which consisted two parts: adamantane‐1,2‐dioxetane and a protective group (aryl‐boronate) at the phenol location and synthesized an activatable chemiluminescence imaging probes by conjugating this linker to an NIR fluorescent dye for imaging H_2_O_2_ activity in a mouse inflammation model. In the presence of H_2_O_2_, the linker was decomposed, resulting in efficient chemical‐resonance‐energy transfer (CRET) between the produced high‐energy intermediate and the conjugated dye, thereby turning on chemiluminescence signals.^[^ ^93^ ^]^ In another work, the same group designed an adamantine‐enol‐ester linker as a precursor of the adamantine‐1,2‐dioxetane linker, which could be specifically activated by ^1^O_2_ to form phenol‐dioxetane and then emit green light in water to detect and image the intracellular ^1^O_2_ produced by a photosensitizer (mTHPC) in HeLa cells.^[^ ^89^ ^]^

However, compared with green light, NIR light has significantly greater ability to penetrate into tissues. In order to obtain more precise and deep‐tissue imaging, in 2020, Shabat's group developed another ^1^O_2_ responsive adamantine‐1,2‐dioxetane linker that contained a dicyano‐methylchromone (DCMC) as an NIR fluorophore at the ortho position of phenol, to construct a near‐infrared chemiluminescence probe (CL‐SO) for monitoring the ^1^O_2_ levels induced by PDT in MCF‐7 cells and corresponding mice model. Upon activation by ^1^O_2_, the linker was oxidized to phenol‐dioxane and spontaneously decomposed into a corresponding excited carbonyl structure in water. Subsequently, the excited state decayed to the ground state accompanied by the production of near‐infrared chemiluminescence and hence perceived ^1^O_2_ imaging.^[^ ^25^ ^]^

**FIGURE 2:**
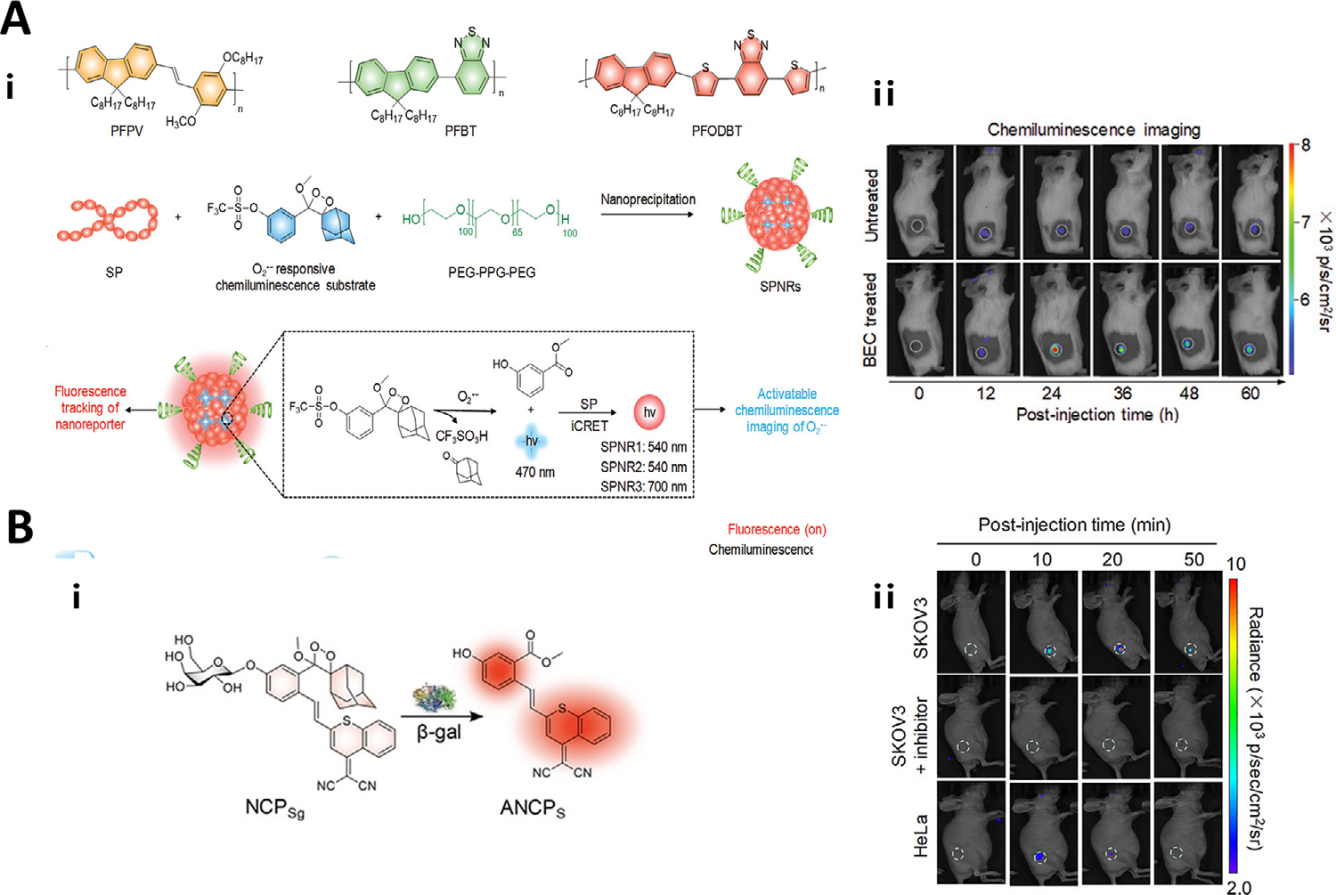
Responsive linkers for chemiluminescence imaging. A) O^2•−^‐responsive linker used for imaging of near‐infrared chemiluminescent. Chemical structures of SPs (PFPV, PFBT, and PFODBT); Schematic illustration of the synthesis of SPNRs via nanoprecipitation; Illustration of the mechanism of O^2•−^ activated chemiluminescence of SPNRs; In vivo chemiluminescence imaging of O^2•−^ in the tumors of living mice after BEC‐mediated immunotherapy. Reproduced with permission.^[^ ^140^ ^]^ Copyright 2019, WILEY‐VCH Verlag GmbH & Co. KGaA, Weinheim. B) β‐gal‐responsive linker used for chemiluminescence detection of β‐gal; Representative NIR chemiluminescent images of living mice. Reproduced with permission.^[^ ^44^ ^]^ Copyright 2020, Wiley‐VCH GmbH.

In addition, the adamantine‐1,2‐dioxetane linker was also used to detect superoxide anion (O^2•−^) which is a significant immunoactivation biomarker for monitoring activation of helper T cells (CD4^+^ T cells) and cytotoxic T cells (CD8^+^ T cells).^[^ ^139^ ^]^ For instance, Cui et al. reported the first O^2•−^‐activatable near‐infrared chemiluminescent reporter that could detect O^2•−^ for real‐time imaging of cancer immunotherapy in the 4T1‐tumor‐bearing mice. The reporter utilized a O^2•−^‐cleavable trifluoro‐methanesulfonate (Tf) caged damantine‐1,2‐dioxetane linker to turn on chemiluminescence signal. Upon reaction with O^2•−^ produced in 4T1‐tumor tissues after S‐(2‐boronoethyl)‐l‐cysteine hydrochloride (BEC)‐mediated immunotherapy, the Tf group was cleaved from the linker, causing the formation of a highly unstable phenolate‐dioxetane derivative, which decomposed spontaneously and released photons with a wavelength of 470 nm. Then, intraparticle chemiluminescence resonance energy transfer (iCRET) from the unstable derivatives to the SP (PFPV, PFBT, and PFODBT) occurred, leading to the generation of chemiluminescence signals at the long wavelength range of the SP (Figure 2A).^[^ ^140^ ^]^ Apart from the ROS‐responsive linkers mentioned above, other linkers such as H_2_O_2_‐responsive luminol,^[^ ^90^ ^]^ peroxyoxalate,^[^ ^141^ ^] 1^O_2_‐responsive thiophene,^[^ ^142^ ^]^ O^2•−^‐responsive imidazopyrazinone^[^ ^111^ ^]^ have been used to construct CLI probe to detect phosphatidylserine (PS)‐positive tumor exosomes, image local inflammation in lymphatic inflammation model of mice, evaluate photodynamic therapy efficacy in MC38 cell tumor‐bearing BALB/c nude mice, and real‐timely detect O^2•−^ in HL‐7702 cells, respectively. Other linkers, such as azanone (HNO)‐responsive azaylide linker,^[^ ^143^ ^]^ H_2_S‐responsive 2,4‐dinitrothiophenol linker,^[^ ^103^ ^]^ GSH‐responsive 2,4‐dinitrobenzenesulfonyl linker,^[^ ^144^ ^]^ have also been reported for activatable CLI probes.

Besides ROS‐responsive linkers, enzyme responsive linkers have also been utilized to construct chemiluminescence probes. For example, Huang et al. used a glycosidic linker to develop a molecular chemiluminescence probe NCPSg for the detection of β‐gal expression levels in SKOV3 (β‐gal‐positive) or HeLa tumor (β‐gal‐negative)‐bearing nude mice. After intratumoral injection of NCPSg, the chemiluminescence intensity of SKOV3 tumor increased gradually due to the cleavage of glycosidic linker overexpressed in the β‐gal‐positive tumor and the subsequent release of DBTP‐phenoxy‐dioxetane (DPD‐S) chemiluminophore. At the same time, 6.5‐fold stronger chemiluminescence intensity was observed in SKOV3 tumor than that in HeLa tumor, confirming the ability of the NCPSg probe to sensitively detect β‐gal activity and differentiate its expression levels in different tumors (Figure 2B).^[^ ^44^ ^]^ Gnaim et al. used a β‐galactose linker that could be cleaved by β‐gal to release a chemotherapeutic agent (monomethyl auristatin E) and a chemiluminescent reporter (dioxetane) to monitor prodrug activation in HEK293 cells and mice bearing β‐gal‐overexpressing tumors.^[^ ^80^ ^]^ Moreover, chemiluminescent probes modified with enzyme‐responsive linkers have also been used to monitor immune cell activity by taking advantage of the corresponding enzymes (e.g. neutrophil elastase, granzyme B). For instance, Huang et al. used a neutrophil elastase‐cleavable linker (VPAA) to design an activatable chemiluminescent probe (BTPDNe) that turned on its chemiluminescence signal in the presence of neutrophil elastase to monitor neutrophils in mouse models of peritonitis and psoriasis.^[^ ^145^ ^]^ Scott et al. used a granzyme B‐cleavable linker (IEPD) to develop a novel chemiluminescence probe for in situ imaging of granzyme B‐mediated killing activity of NK cells against MDA‐MB‐231 cells.^[^ ^72^ ^]^

In addition, many other enzymes‐responsive linkers have been used for the detection and chemiluminescence imaging of enzyme‐related diseases, such as carbapenemase‐responsive carbapenem linker,^[^ ^73^ ^]^ fibroblast activation protein‐alpha (FAPα) ‐responsive dipeptide linker (glycine‐proline),^[^ ^74^ ^]^ γ‐glutamyl transpeptidase (GGT)‐responsive linker (γ‐Glu),^[^ ^75^ ^]^ leucine aminopeptidase (LAP)‐responsive linker (l‐leucine),^[^ ^76^ ^]^ neuraminidase‐responsive linker (*N*‐acetylneuraminic acid),^[^ ^77^ ^]^ quinone oxidoreductase‐1 (NQO1)‐responsive linker (trimethyl‐locked quinone)^[^ ^78^ ^]^ and so on.

#### 3.1.3 Dual‐locked optical imaging

Unlike the single biomarker responsive linkers, the use of two different responsive linkers that respond to two biomarkers respectively have been explored for dual‐locked optical imaging which can be specifically activated by different biomarkers and then produce optical signals for real‐time imaging and detecting multiple disease associated analytes. This kind of dual‐responsive probes can greatly enhance the accuracy of disease diagnosis through the response toward two elevated biomarkers.^[^ ^146^ ^]^ Currently, some dual responsive imaging probes such as dual enzyme responsive probes, ROS and enzyme dual‐responsive probes, light and enzyme dual‐responsive probes, and so on have been applied for dual‐locked optical imaging of cancers,^[^ ^123^, ^124^, ^125^, ^126^, ^127^ ^]^ hepatopathy^[^ ^128^, ^129^ ^]^ and other diseases.^[^ ^27^, ^147^, ^148^, ^149^ ^]^

**FIGURE 3:**
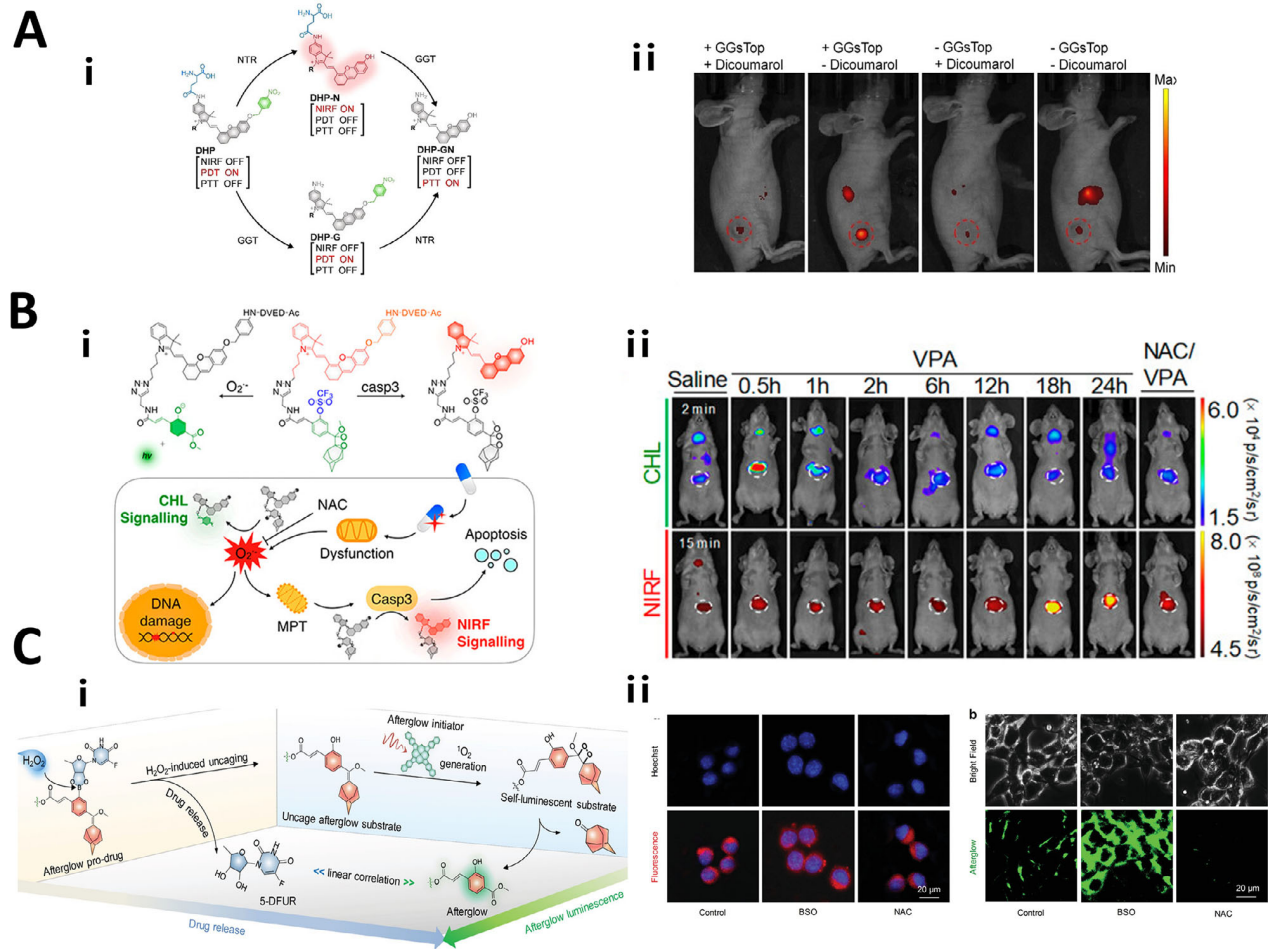
Responsive linkers for dual‐locked optical imaging. A) Nitroreductase (NTR) and γ‐glutamyl transferase (GGT)‐responsive linkers used for imaging of tumor and auto‐regulated PDT‐PTT; In vivo real‐time NIRF imaging of tumor in mice. Reproduced with permission.^[^ ^151^ ^]^ Copyright 2022, Wiley‐VCH GmbH. B) ROS and caspase‐3‐responsive linkers used for detecting the sequential upregulation of O^2•−^ and caspase‐3. Duplex Imaging Mechanism of CFR; Real‐time duplex imaging of DIH in living mice after i.v. injection of CFR. Reproduced with permission.^[^ ^153^ ^]^ Copyright 2019, American Chemical Society. C) H_2_O_2_ and O^2•−^‐responsive linkers to monitor drug release. H_2_O_2_‐induced activation of prodrug and afterglow luminescence for APtN; In vitro fluorescence and afterglow imaging of APtN in 4T1 cells. Reproduced with permission.^[^ ^156^ ^]^ Copyright 2019, WILEY‐VCH Verlag GmbH & Co. KGaA, Weinheim.

In the tumor microenvironment, overexpression of enzymes such as γ‐glutamyl transferase, nitroreductase and caspase, is accompanied with tumor invasion.^[^ ^150^ ^]^ Different linkers that can respond to the cancer‐related enzymes can be integrated together in the same imaging probe for precisely imaging cancer and detecting therapeutic efficacy, with improved accuracy. For instance, Wei et al. utilized two enzyme‐responsive linkers (nitroreductase (NTR)‐responsive 4‐methylenecyclohexa‐2,5‐dien‐1‐imine linker and γ‐glutamyl transferase (GGT)‐responsive γ‐glutamate linker) to modify a hemicyanine (DHCy) molecule and produced a dual‐locked activatable photo‐theranostic probe (DHP) for near‐infrared fluorescence (NIRF) imaging with auto‐regulated PDT‐PTT cervical cancer therapy. The DHP was non‐fluorescent because of the intramolecular charge transfer (ICT) effect caused by caging the hydroxyl group with the NTR‐cleavable linker. Once the NTR responsive linker was cleaved, highly fluorescent DHP‐N was generated. Following cleavage of the glutamate linker by GGT, the final product DHP‐GN with active PTT was obtained. Thus, this design combined activatable real‐time NIRF imaging with automatic regulation of tumor‐specific PDT‐PTT to provide an intelligent strategy for precise phototheranostics (Figure 3A).^[^ ^151^ ^]^ Wang et al. used a GGT‐responsive γ‐glutamate linker and a caspase‐1‐responsive *N*‐acetyl‐Tyr‐Val‐Ala‐Asp (YVAD) linker to link a hemicyanine NIRF signaling moiety and developed a dual‐locked NIR fluorescence probe (DTAP) for predicting cancer therapeutic efficacy via real‐time imaging of intratumoral pyroptosis in 4T1‐tumor‐bearing mice.^[^ ^152^ ^]^

ROS and enzyme dual‐responsive linkers have also been conceived in the design of dual‐locked optical imaging probes. For example, H_2_O_2_‐responsive borate linker and tyrosinase (TYR)‐responsive 3‐hydroxybenzyloxy linker were reported to construct a dual‐locked near‐infrared fluorescent probe (MB‐m‐borate) for diagnosis of melanoma.^[^ ^29^ ^]^ The locking borate and 3‐hydroxybenzyloxy linkers could be cleaved by high level of H_2_O_2_ and tyrosinase in melanoma cells, respectively, resulting in the nonfluorescent MB‐m‐borate to generate fluorophore methylene blue (MB) which could emit NIR fluorescence with a wavelength of 684 nm. Furthermore, ROS and enzyme dual‐responsive linkers have been applied to detect drug‐induced hepatotoxicity (DIH) and contrast‐induced acute kidney injury (CIAKI). Chen et al. designed a unimolecular chemo‐fluoro‐luminescent reporter (CFR) for duplex imaging of DIH by using a O^2•−^‐responsive trifluoromethanesulfonate (TF) linker and a caspase‐3‐responsive DEVD linker. The CFR was initially dual‐locked because the DEVD linker caged a NIRF moiety hemicyanine unit (CyU) and reduced the electron‐supplying capacity of oxygen atoms. Meanwhile, a CHL moiety dioxetane was inhibited by caging phenol with the O^2•−^‐cleavable TF linker. Valproic acid (VPA) is a widely prescribed antiepileptic drug. The high dosage of VPA was used to induce oxidative stress and increase the generation of ROS. *N*‐acetyl l‐cysteine (NAC) was used to scavenge cellular ROS, prevent oxidative stress and subsequently inhibit apoptosis. After the two linkers were cleaved, the near‐infrared fluorescence (NIRF) and chemiluminescence (CHL) signal of resulted product was turned on, allowing for detecting the sequential upregulation of caspase‐3 and O^2•−^ during the progression of DIH (Figure 3B).^[^ ^153^ ^]^ Huang et al. utilized a O^2•−^‐responsive trifluoromethanesulfonate linker and a *N*‐acetyl‐β‐d‐glucosaminidase (NAG)‐responsive *N*‐acetyl‐β‐d‐glucosamine linker to cage chemiluminescent phenoxy‐dioxetane and a NIRF hemi‐cyanine moiety to construct an activatable duplex reporter for duplex‐imaging and monitoring the progression of CIAKI.^[^ ^154^ ^]^

In addition, photocleavage of linkers induced by light have been applied to design dual‐locked optical imaging probes. Thiel et al. reported a light‐responsive O‐nitrobenzyl linker and nitroreductase‐responsive nitro linker to design a fluorescent probe for imaging nitroreductase activity within mitochondria in mammalian cells. The nitro linker of this diazoindole‐modified rhodamine analogue probe would extract sufficient electron density under 350 nm light irradiation and generate a non‐fluorescent product. However, enzymatic cleavage by nitroreductase produced amino‐substituted electron‐rich xanthan, allowing for the imaging of nitroreductase activity.^[^ ^155^ ^]^ Furthermore, NIR Light can interact with photosensitizers to produce singlet oxygen (^1^O_2_), which can in turn react with specific linkers to enable chemiluminescence imaging. For example, He et al. used a H_2_O_2_‐NIR light dual‐responsive linker to develop an organic afterglow protheranostic nanoassembly (APtN) to image a prodrug (5′‐deoxy‐5‐fluorouridine, 5‐DFUR) activation. Upon accumulation of APtN in 4T1 tumor‐bearing mice, the H_2_O_2_‐responsive boronic ester linker was cleaved by tumor‐upregulated H_2_O_2_, allowing the release of 5‐DFUR. Then, the ^1^O_2_‐responsive adamantylidene enol ether linker could be cleaved by ^1^O_2_ produced by the photosensitizer (silicon 2,3‐naphthalo‐cyanine bis(trihexylsilyloxide)) upon NIR light (808 nm) irradiation at tumor sites to produce 1,2‐dioxetane chemiluminescence substrate to image and monitor drug release (Figure 3C).^[^ ^156^ ^]^

Another biomarker that can be used to create dual‐responsive linkers is hydrogen sulfide.^[^ ^157^ ^]^ For example, Zhang et al. used a H_2_S‐responsive NBD (7‐nitro‐1,2,3‐benzoxadiazole) amines linker and a quinine oxidoreductase 1 (hNQO1)‐responsive quinone propionic acid (Q_3_PA) linker to design a dual‐responsive probes for the detection of both endogenous H_2_S and hNQO1 activity in live cells. Only dual cleavage of both the NBD and Q_3_PA linker in the presence of H_2_S and hNQO1 in HT29 and HepG2 cells can produce fluorescence signal for the simultaneous imaging of the two biomarkers.^[^ ^158^ ^]^ Ou et al. utilized a H_2_S‐responsive ‐N_3_ linker and a phosphatase‐responsive phosphate linker to develop a dual‐reactive fluorescent probe (N_3_‐CR‐PO_4_) for measuring of H_2_S level and phosphatase activity in HeLa cells.^[^ ^65^ ^]^

Compared with single‐factor activatable probes, dual‐locked probes have the advantages of avoiding non‐specific activation and ‘false positive’ response in complex environments. However, the most important obstacle for many dual‐ locked systems is the elimination of potential crosstalk, which means the use of two similar fluorescence channels can result in emission crosstalk during bioimaging. Therefore, using two independent optical channels or the combination of chemiluminescence with fluorescence is required.

### 3.2 In magnetic resonance imaging (MRI)

Magnetic resonance imaging (MRI) is a noninvasive imaging technique widely used for disease diagnosis due to its good spatial resolution and tunable soft tissue contrast. However, due to the highly abundant water molecules in the body, the signal‐to‐background ratio of MRI is very low, which leads to the inherent low sensitivity of MRI.^[^ ^159^ ^]^ Contrast agents (CAs) have been widely used to increase contrast effect between tumor tissues and normal tissues by reducing the relaxation time of water protons, thus improving the signal‐to‐background ratio. Depending on the relaxation of the signal, MRI contrast agents can be divided into two categories: *T*_1_ weighted MRI contrast agents and *T*_2_ weighted MRI contrast agents. *T*_1_ weighted contrast agents shorten the longitudinal relaxation time (*T*_1_) of water protons, resulting in production of brighter (*T*_1_ weighted) MR images. Gadolinium (Gd) chelate is one of the most frequently used *T*_1_ weighted MRI CAs and can improve the contrast efficacy by shortening the longitudinal relaxation time of water protons around Gd^3+^ ions.^[^ ^160^ ^]^ Other paramagnetic metal ions chelates such as manganese ion (Mn^2+^) are also used for *T*_1_ weighted MRI CAs. On the contrary, *T*_2_ weighted contrast agents shorten the transverse relaxation time (*T*_2_) of water protons, which translates to darker (*T*_2_ weighted) MR images. These reagents are typically superparamagnetic iron oxide nanoparticles (SPIONs).^[^ ^161^ ^]^ The effect of *T*_1_ and *T*_2_ weighted CAs can be determined by its longitudinal relaxivity (*r*_1_) and transversal relaxivity (*r*_2_), respectively, which describes the efficiency that a CA can affect the relaxation rate of water protons and is measured in per millimolar per second (mm ^−1^ s^−1^).^[^ ^159^ ^]^

Although MRI CAs enhance the contrast between tissues to some extent, the intrinsic low sensitivity of MRI is still a problem. To overcome this issue, there has been an increasing interest in the design of targeted CAs, which allow selective accumulation of CAs at target sites to increase the signal‐to‐background ratio. However, these target MRI CAs typically only obtain anatomical and functional information.

The introduction of responsive linkers in MRI CAs can not only improve the sensitivity of CAs but also achieve molecular MR imaging, allowing visualization of disease‐related biomarkers and understanding biological and therapeutic processes. Recently, tumor microenvironment (TME) responsive linkers such as enzyme‐responsive, redox‐responsive linkers, pH‐responsive linkers and so on have been applied to design responsive MR probes, which can amplify the MRI signal based on the specific cleavage of responsive linkers in the TME and provide more accurate information for precise diagnosis of cancer at a molecular level. Therefore, in this section, we summarize the responsive linkers used in *T*_1_ and *T*_2_ weighted MRI, respectively.

#### 3.2.1 Responsive linkers for *T*_1_ weighted MRI

By using responsive linkers, researchers have designed various activatable *T*_1_ weighted MRI CAs which can enhance the *T*_1_ weighted MR signal once the linkers are cleaved under specific enzyme, redox, pH conditions etc. The MR signal enhancement is ascribed to either the increase of hydration number (*q*), or the tumbling rate (*τ*_R_), which correspond to an increased *r*_1_.^[^ ^162^ ^]^ Here, we summarize responsive linkers that are able to mediate *q* and *τ*_R_ values for *T*_1_ weighted MR imaging with great specificity.

**FIGURE 4:**
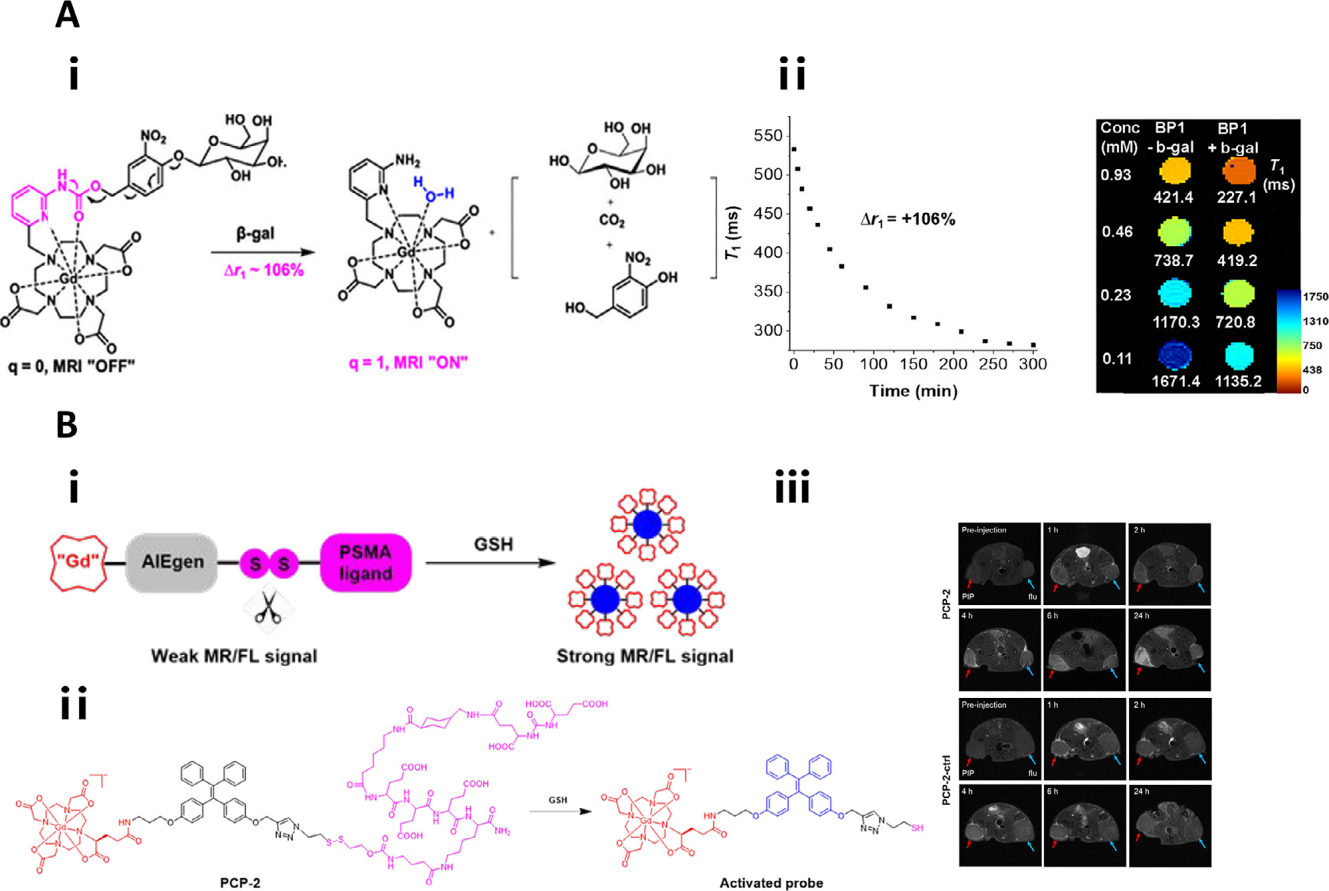
Responsive linkers used in *T*_1_ weighted MRI. A) β‐gal‐responsive linkers increased *q* value with an increased *r*_1_. In the presence of β‐gal, the sugar moiety is enzymatically removed and *q* increases to 1 with a significant increase in the observed MR signal; Time‐dependent *T*_1_ decreases of the agents in the presence of β‐gal; *T*_1_‐weighted phantoms of the agents. Reproduced with permission.^[^ ^82^ ^]^ Copyright 2023, American Chemical Society. B) GSH‐responsive linkers slowed tumbling rate with an increased *r*_1_. Chemical Structures of PCP‐2 and its activated product; PCP‐2 and PCP‐2‐ctrl MR imaging of PC3pip (red arrow) and PC3flu (blue arrow) tumor xenografts. Reproduced with permission.^[^ ^108^ ^]^ Copyright 2021, American Chemical Society.

The *T*_1_ weighted MR contrast agents modified with enzyme‐responsive linkers have been designed to respond to β‐galactosidase, cathepsin‐B, caspase, matrix metalloprotease‐2 (MMP‐2), alkaline phosphatase (ALP), and so forth. For instance, Lilley and coworkers used a galactose linker to detect β‐gal activity in transgenic LacZ mice expressing β‐gal. The linker underwent hydrolysis in the presence of β‐gal that provided an open coordination site for H_2_O molecules to bind to Gd(III), and an effective *q*‐modulation as well as significant enhancement of *T*_1_ weighted MR signal was observed in the transgenic LacZ mouse model compared to the non‐transgenic native control mice.^[^ ^45^ ^]^ In another work, the group also used the galactose linker to develop a β‐gal‐responsive MRI probe to monitor adeno‐associated virus (AAV) gene therapy in a murine model of GM1‐gangliosidosis. Upon activation by β‐gal, the galactose linker was cleaved and exposed Gd(III) to water, thus enhancing *q* values and turning on the MR signal for tracking β‐gal activity after AAV gene therapy.^[^ ^81^ ^]^ To increase the imaging sensitivity, the same group recently also exploited the galactose linker to attach a new pendant carboxylate ligand‐based Gd(III) contrast agent for visualizing β‐gal activity. The gradual decrease of *T*_1_ along with the incubation periods indicated that the β‐gal enzyme had turned on the signal. Furthermore, it showed that the 39.2 and 89.0% increases of 1/*T*_1_ were observed after 1 and 5 h. Upon cleavage, the agent's *q* value increased from 0 to 1 and showed more than 5‐fold increase in Δ*r*_1_ (Δ*r*_1_ = 106%) in comparison with their previously developed activatable carboxylate ligand‐based Gd(III) MR agents (Figure 4A).^[^ ^82^ ^]^

In addition, cathepsin‐B responsive linkers have also been exploited to construct activatable *T*_1_ weighted MRI agents. The GFLG linker was used by Cai's group to fabricate a branched polymeric PTX‐Gd‐based nanoparticles (BP‐PTX‐Gd NPs) for to image PTX treatment effectiveness to 4T1 tumor‐bearing mice. The introduction of pHPMA polymer containing a stable GFLG linker greatly enhanced the MRI contrast compared to Gd‐DTPA (*r*_1_ value: 8.6 mm ^−1^ s^−1^ vs. 3.6 mm ^−1^ s^−1^). Furthermore, cleavage of the GFLG linker led to the release of PTX from the NPs, and this treatment process was closely associated with an increase of the *T*_1_ values at the tumor site.^[^ ^37^ ^]^ Li et al. employed the caspase‐3/7 cleavable DEVD linker to attach a DOTA‐Gd(III) chelate‐tetraphenylethylene AIEgen (Gad‐AIE) to form a caspase probe 1 (CP1) to image apoptosis. The CP1 probe remained water soluble and showed a low MR signal in the absence of caspase‐3/7. In contrast, the cleavage of DEVD by caspase‐3/7 resulted in the aggregation of the remaining Gd‐AIE residues, which could prolong *τ*_R_ with enhanced MR signal to monitor therapy‐induced HeLa cells apoptosis.^[^ ^51^ ^]^ Other enzyme‐responsive linkers such as matrix MMP‐2‐responsive GPLGVRG linker,^[^ ^58^ ^]^ ALP‐responsive phosphate (−PO_3_H) linker^[^ ^163^ ^]^ have also been applied to design *T*_1_ MRI contrast agents for in vivo imaging of enzyme activity.

Apart from abnormally expressed enzymes, the GSH‐responsive linkers were also exploited in the design of *T*_1_ weighted MRI CAs. For example, the disulfide linker was utilized by Li and coworkers to conjugate a PSMA‐targeting ligand with a Gd (III)‐TPE to form a GSH activatable Gd(III) based MR CAs (PCP‐2) for PSMA imaging. Once entering PSMA positive prostate cancer cells, the disulfide linker was cleaved by intracellular GSH and the probe was subsequently self‐assembled into nanoaggregates with 53.0% increase in *r*_1_ values due to lower tumbling rate (*τ*_R_), providing enhanced MR signal for PSMA positive tumor (Figure 4B).^[^ ^108^ ^]^ In addition, GSH‐responsive disulfide linker can switch contrasting enhancement from *T*_2_ to *T*_1_. Cao et al. exploited this concept by synthesizing a GSH activatable *T*_1_‐switchable MR contrast agents (ICNs‐RGD) that encapsulated ESIONP‐CA in disulfide linker cross‐linked poly(CBMA) nanogels equipped with c(RGD) ligand. After GSH activation, the *r*_1_ value increased slightly from 5.56 to 7.40 mm ^−1^s^−1^, but the *r*_2_ value decreased sharply from 103.01 to 14.36 mm ^−1^s^−1^, indicating that ICNs‐RGD had the potential to become an effective *T*_1_ weighted MRI CA.^[^ ^109^ ^]^

By taking advantage of the acidic tumor microenvironment, some pH‐responsive linkers or substances have been explored to enhance the *T*_1_ MRI signal of CAs. For example, Shen et al. used an acid‐responsive β‐thiopropionate linker to graft poly(ethylene glycol) methyl ether (mPEG) onto *T*_1_ weighted exceedingly small magnetic iron oxide nanoparticles (ES‐MIONs), preventing the targeting ligand dimeric RGD peptide (RGD2) from binding to normal cells with non‐specific receptors. However, under weakly acidic tumor conditions, the β‐thiopropionate linker was cleaved, leading to the detachment of mPEG from the nanoparticles and exposure of the hidden RGD2 toα_v_β_3_‐expressing U‐87 MG cancer cells. The enhanced aggregation of the nanoparticles at the target site benefited the *T*_1_‐weighted MR imaging (*r*_1_ value was up to 8.80 mm ^−1^ s^−1^ at 1.5 T, ΔSNR is 203.4 ± 15.1% at 12 h postinjection).^[^ ^118^ ^]^ In addition, some materials with high pH‐responsive degradability such as amorphous porous manganese phosphate (PMP) nanoparticles^[^ ^164^ ^]^ and manganese silicate nanoparticles (MnSNs)^[^ ^165^ ^]^ have been reported to amplify *T*_1_ signal at tumor sites through Mn^2+^ release under acidic tumor environment.

#### 3.2.2 Responsive linkers for *T*_2_ weighted MRI

For the *T*_2_ weighted MRI CAs, the improvement of *T*_2_ relaxation upon cleavage of the linkers is due to the formation of CAs aggregates to slow down their tumbling time or improve their superparamagnetism. Responsive linkers show great potential in designing highly sensitive *T*_2_ weighted MRI CAs for precise diagnosis of disease at a molecular level.

Some enzyme‐responsive linkers have been developed to slow the tumbling time through in situ formation of CAs aggregates, thus leading to higher *r*_2_ values and enhanced *T*_2_ weighted MR signal. For example, Liang and coworkers employed an alkaline phosphatase (ALP)‐responsive Nap‐FFFYp linker to design a hydrogelator Nap‐FFFYp‐EDA‐OTA(Gd) (1P) which could self‐assembled into gadolinium nanofibers upon ALP catalysis, leading to decreased tumbling time as well as increased *T*_2_‐weighted MR signal (33.9% enhancement of the *r*_2_ value) for imaging ALP activity in ALP‐overexpressing HeLa tumors‐bearing nude mice.^[^ ^66^ ^]^ Another Gd‐based *T*_2_ weighted contrast agent was exploited in a similar strategy by using a γ‐glutamyltranspeptidase (GGT)‐responsive γ‐glutamyl linker, yielding Glu‐Cys(StBu)‐Lys(DOTA‐Gd)‐CBT for *T*_2_‐weighteded MRI. Cleavage of the γ‐glutamyl linker caused the formation of Gd nanoparticles, which increased *r*_2_ values (from 5.79 to 25.1 mm ^−1^s^−1^) and *T*_2_ weighted MR contrast allowing for imaging of GGT‐related cancers.^[^ ^166^ ^]^

**FIGURE 5:**
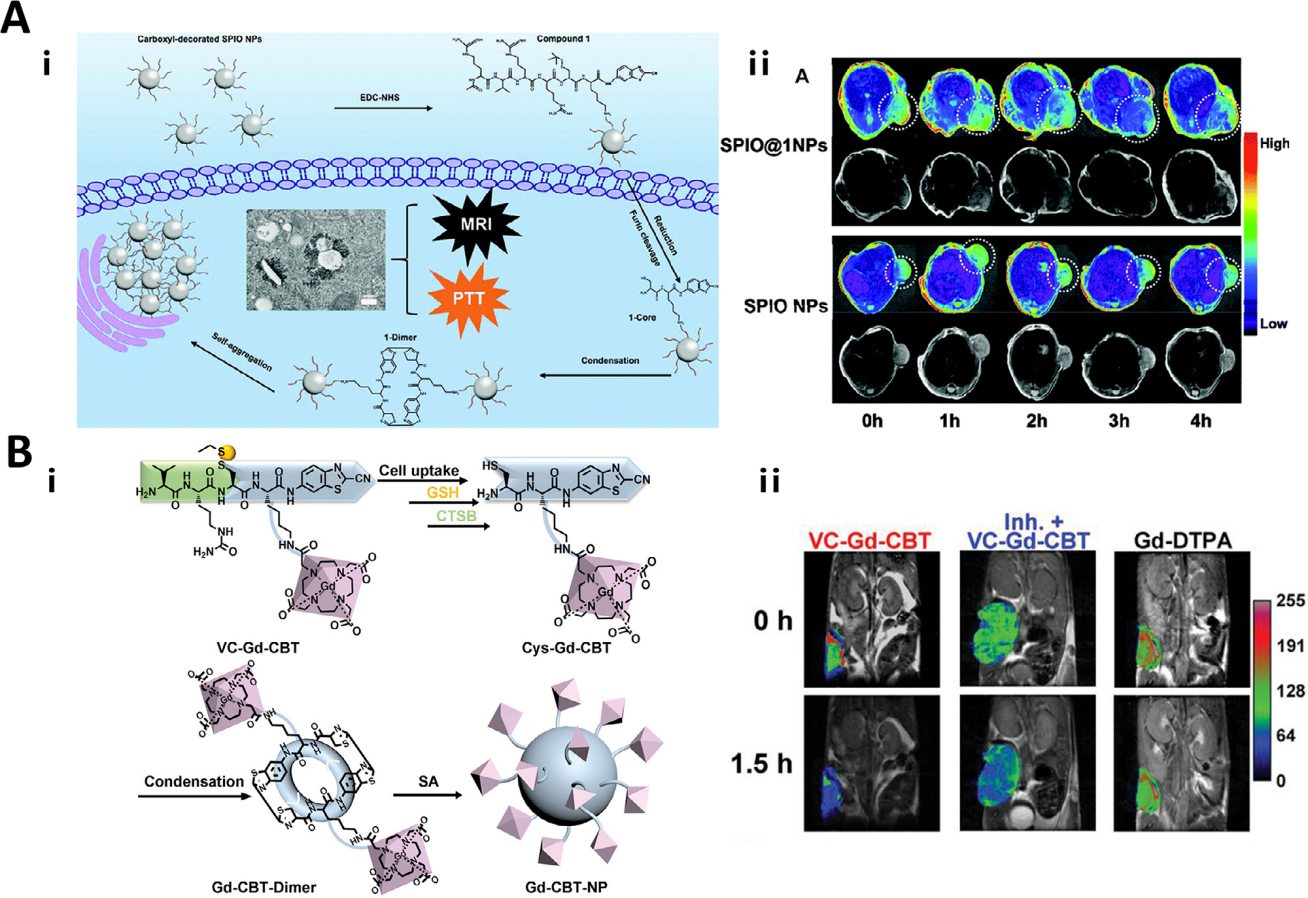
Responsive linkers used in *T*_2_ weighted MRI. A) Furin‐responsive linkers triggered aggregation of SPIO@1NPs with increased transverse relaxivity. Chemical structures of SPIO@1NPs and schematic illustration of SPIO@1NPs intracellular aggregation; Enhancement of *T*_2_‐weighted imaging contrast is due to the maximum aggregation of SPIO@1NPs in tumors. Reproduced with permission.^[^ ^63^ ^]^ Copyright 2020, Royal Society of Chemistry. B) Cathepsin B‐responsive linkers triggered the formation of Gd nanoparticles with much shorter *T*_2_ relaxation time. CTSB‐guided intracellular formation of Gd‐CBT‐NPs for enhanced *T*_2_‐weighted MRI. After VC‐Gd‐CBT enters CTSB‐overexpressing cancer cells, it undergoes cleavage of the peptide by CTSB and reduction by GSH to generate the cleaved Cys‐Gd‐CBT, then self‐assemble to produce Gd‐CBT‐NP; *T*_2_‐weighted coronal MR images of MDA‐MB‐231 tumor‐bearing mice. Reproduced with permission.^[^ ^40^ ^]^ Copyright 2023, Elsevier Ltd.

Encouraged by above studies, a furin‐responsive TFMB‐Arg‐Val‐Arg‐Arg (TFMB‐RVRR) linker was designed by the same group to develop IONP@1 for *T*_2_ MRI imaging of MDA‐MB‐468 tumor‐bearing zebrafish. The cleavage of TFMB‐RV gave rise to the formation of IONP aggregates which gave an approximately 81.9% increase in *r*_2_ values.^[^ ^84^ ^]^ The same RVRR linker was also used to design Ac‐RVRR‐Cys(StBu)‐Lys(SPIO)‐CBT (SPIO@1NPs) to image MDA‐MB‐468 tumors. The cleavage of RVRR linker aggregated the SPIO@1NPs, leading to about 63.96% increase of *r*_2_ values than the NPs in the monodispersed state (Figure 5A).^[^ ^63^ ^]^ Similarly, Liang's group functionalized the nanosystem with a DVED linker to produce Fe_3_O_4_@1 NPs for *T*_2_ weighted MR imaging of caspase‐3/7 activity. Upon cleavage by caspase‐3/7 in apoptotic HepG2 cells or tumors, the aggregation of Fe_3_O_4_@1 NPs largely shortened the transverse relaxation time (*T*_2_) and induced approximately 65.2% enhancement of *r*_2_ values, indicating the potential of the DEVD linker for enhancing *T*_2_ imaging of tumor apoptosis.^[^ ^52^ ^]^ The cathepsin B‐responsive VC dipeptide linker has also been reported for activatable *T*_2_ weighted MRI CAs. A VC‐Cys(SEt)‐Lys(DOTA‐Gd)‐CBT (VC‐Gd‐CBT) CAs was designed and showed that cathepsin B was able to trigger the formation of Gd nanoparticles along with much shorter *T*_2_ relaxation time (217.2 ms) compared to the unassembled VC‐Gd‐CBT (361.2 ms) and largely increased *T*_2_ weighted contrast in cathepsin‐overexpressing MDA‐MB‐231 tumors at 9.4 T (Figure 5B).^[^ ^40^ ^]^

MMP‐9‐responsive linkers are another category in the design of activatable *T*_2_ weighted CAs. Schellenberger et al. designed an MMP‐9‐activatable protease‐specific iron oxide particles (MMP‐9‐PSOP) using the PRQITA linker, the cleavage of which could aggregate the PSOP to switch from a low‐relaxivity stealth state to large clustered aggregates with increased *r*_2_ relaxivity.^[^ ^62^ ^]^ In another report, Gallo et al. used a PLGMWSR‐OH linker to design CXCR4‐targeted iron oxide nanoparticles (IONPs) which could be activated by MMP‐9 and self‐assembled to give a *T*_2_ signal enhancement by about 160% in U87.CD4.CXCR4 tumor.^[^ ^57^ ^]^

In addition, some pH‐responsive linkers have also been used to improve the *T*_2_ MRI signals of CAs. For example, Yang et al. used a pH‐responsive methyloxy‐poly(ethylene glycol)‐*block*‐poly[dopamine‐2‐(dibutylamino) ethylamine‐l‐glutamate] (mPEG‐b‐P(DPA‐DE) LG) copolymer to encapsulate Fe_3_O_4_ nanoparticles as pH‐responsive CAs for *T*_2_ weighted MRI. The pH‐responsive mPEG‐b‐P(DPA‐DE) LG micelles could rapidly release Fe_3_O_4_ nanoparticles in response to an acidic environment in a cerebral ischemia disease rat model, allowing excellent *T*_2_ weighted MR diagnosis of cerebral ischemia diseases.^[^ ^119^ ^]^ Crayton and colleagues attached a pH‐responsive polymer glycol chitosan (GC) to the surface of dextran‐stabilized SPIO nanoparticles to create a pH‐responsive *T*_2_‐weighted MR CAs. At physiological pH, the nanoparticles had a neutral or negative surface charge with extended blood circulation, but could be protonated at an acidic condition with the surface charge converted progressively positive, resulting in enhanced nanoparticle accumulation in the T6‐17 tumor bearing nude mice and robust *T*_2_‐weighted contrast.^[^ ^24^ ^]^

Other responsive linkers such as ROS‐responsive thioketal linker,^[^ ^167^ ^]^ redox‐responsive disulfide linker,^[^ ^168^, ^169^ ^]^ and so on have also been used to develop responsive *T*_2_ weighted MRI CAs. These examples demonstrate the advantages of responsive linkers and provide a platform for developing responsive *T*_1_ or *T*_2_ weighted MRI contrast agents with higher sensitivity and contrast efficacy.

### 3.3 In photoacoustic imaging (PAI)

Photoacoustic imaging (PAI) is a three‐dimensional hybrid imaging modality that integrates optical and acoustic imaging, which has broad prospects in molecular imaging due to its high sensitivity, imaging depth, and resolution.^[^ ^170^ ^]^ PAI uses light to stimulate the production of acoustic waves, that after excitation of a chromophore with a pulsed laser, the absorbed energy is converted to heat, which results in rapid thermoelastic expansion in biological tissues to generate a detectable ultrasound signal, and subsequently the ultrasound signal will be reconstructed into PA images by an ultrasound transducer.^[^ ^171^ ^]^ Although PAI combines the advantages of high sensitivity and high penetration depth, the translation of traditional PAI probes still faces many challenges, such as background interference of endogenous chromophores, low imaging specificity, and inability to actively detect important pathologically relevant biomarkers, and so on.^[^ ^170^ ^]^ Fortunately, the construction of reactive PAI probes using responsive linkers supports the specific biomarkers detection and reliable imaging of biological events from the molecular level. Here, we summarize a series of responsive linkers used in PAI probes.

#### 3.3.1 Redox‐responsive linkers used in PAI

**FIGURE 6:**
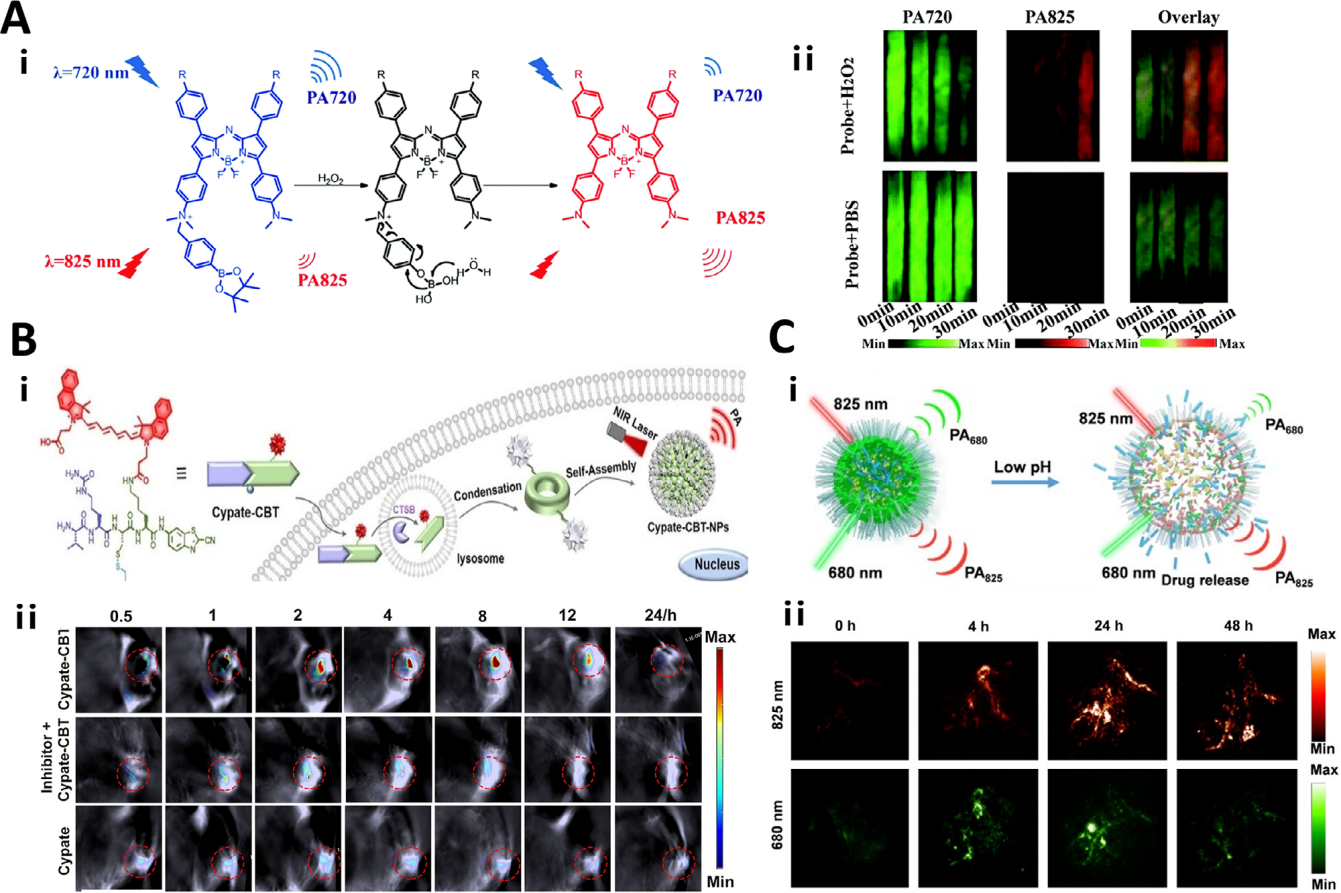
Responsive linkers used photoacoustic imaging. A) H_2_O_2_‐responsive linker used for PA imaging of H_2_O_2_. In the presence of H_2_O_2_, the boronic acid ester group is interrupted to recover the Aza‐Bodipy (red), and this induces a weaker PA signal at 720 nm and stronger PA signal at 825 nm; In vitro ratiometric PA images in the absence and presence of H_2_O_2_. Reproduced with permission.^[^ ^96^ ^]^ Copyright 2018, Royal Society of Chemistry. B) Cathepsin B‐responsive linker used for PA imaging of CTSB activity. CTSB‐triggered self‐assembly of Cypate‐CBT‐NPs for photoacoustic imaging of CTSB activity; In vivo PA images of nude mice bearing MDA‐MB‐231 tumors injected with Cypate‐CBT. Reproduced with permission.^[^ ^41^ ^]^ Copyright 2021, Wiley‐VCH GmbH. C) pH‐responsive linker used for PA imaging of DOX release. At low pH, the HPDI will be protonated (pink), inducing a loosened nanostructure to trigger the release of DOX accompanied by PA signals vanishing at 680 nm. Meanwhile, the chemical structure of IR825 and its characteristic PA signal at 825 nm retain the same. Therefore, the DOX release could be monitored by a ratiometric PA imaging at PA_825_/PA_680_; In vivo PA imaging of tumor pH. Reproduced under terms of the CC‐BY NC 3.0 license.^[^ ^132^ ^]^ Copyright 2017, The Authors.

A series of activatable PAI probes with high‐sensitivity and specificity for detecting ROS, RNS, and RSS have been developed. For example, Lu et al. attached a H_2_O_2_‐responsive benzeneboronic acid pinacol ester linker to an Oligo(ethylene glycol) (OEG) modified NIR absorbing Aza‐BODIPY derivative to form OEG‐Aza‐BODIPY‐BAPE probe. In the presence of H_2_O_2_, the benzeneboronic acid ester linker was cleaved to recover the Aza‐BODIPY(red), inducing a weaker PA signal at 720 nm and a stronger PA signal at 825 nm, and it was used for ratiometric PA imaging of H_2_O_2_ in A549 xenografted tumor bearing mice (Figure 6A).^[^ ^96^ ^]^ Weber et al. utilized a H_2_O_2_‐responsive aryl boronate ester linker to connect a 2‐deoxyglucose modified heptamethine carbocyanine dye scaffold to synthesize a capped PAI probe JW41. After injecting the JW41 probe into MDA‐MB‐231 tumor‐bearing mice, the aryl boronate ester linker could be cleaved in the presence of H_2_O_2_, converting the JW41 to a uncapped dye IW35 with an increase in PA signal between 700 and 810 nm.^[^ ^94^ ^]^ Other ROS‐responsive linkers, such as O^2•−^‐responsive ortho‐phenolic hydroxyl linker,^[^ ^172^ ^]^ 2,6‐di‐t‐butyl‐4‐methylphenol (BHT) linker,^[^ ^173^ ^]^ and ClO^−^‐responsive semiconducting oligomer amphiphile (SOA) linker^[^ ^174^ ^]^ have also been used in activatable PAI probes.

For RNS detection with PAI, the NO and ONOO^−^‐responsive linkers were used. For instance, Reinhardt's group incorporated a NO‐responsive *o*‐aminophenol linker to the aza‐BODIPY dye to produce an APNO‐5 probe, which underwent *N*‐nitrosation to form a concomitant 91 nm hypsochromic, enabling a 1.9‐fold increase in PA signal at 680 nm and a 1.3‐fold ratiometric turn‐on compared to a saline control in a murine lipopolysaccharide (LPS)‐mediated inflammation model.^[^ ^112^ ^]^ More recently, they used the same linker to attach an optimized boron‐azadipyrromethene (aza‐BODIPY) dye to synthesize a SR‐APNO‐3 probe for PA imaging of cancer‐derived NO in an intramuscular lipopolysaccharide‐induced inflammation mice model. The linker went through *N*‐nitrosation to produce a 4.4‐fold increase in the ratiometric PA imaging.^[^ ^175^ ^]^ Furthermore, real‐time imaging of ONOO^−^ in tumors is imperative to understand its underlying mechanism and optimize therapeutic interventions. Zhang et al. attached a ONOO^−^‐responsive bulky borane linker to a boron‐dipyrromethene dye (BBD) and doped it with organic semiconducting nanoprobes (OSNs) for PA imaging of ONOO^−^ in the 4T1 xenograft tumor. The bulky borane linker could be degraded upon reacting with ONOO^−^, and ΔPA_750_/ΔPA_680_ gradually increased and reached its maximum at 4 h post injection which was 2.6‐fold higher than the NAC‐treated mice with reduced ONOO^−^ level.^[^ ^102^ ^]^

The rise of H_2_S in cancer and other diseases emphasizes the significance of specific detection and monitoring of H_2_S. Chen et al. used a H_2_S‐responsive benzoic ester linker to design a meso‐benzoyloxyltricarboheptamethine cyanine HS‐CyBz for PA imaging of H_2_S.^[^ ^104^ ^]^ Ma et al. used a H_2_S‐responsive AzHD linker to construct nanoprobe AzHD‐LP for PA imaging of H_2_S in the HCT116 colon tumor‐bearing mice.^[^ ^105^ ^]^ As for GSH, Yin et al. exploited GSH‐responsive disulfide linker to develop IR806‐PDA for ratiometric PAI of GSH in HeLa‐tumor bearing nude mice.^[^ ^26^ ^]^

#### 3.3.2 Enzyme‐responsive linkers used in PAI

Enzyme‐responsive linkers are also applied to develop activatable PAI probe. For example, Yao et al. used an ALP‐responsive phosphate ester linker to connect a NIR fluorescent hemicyanine dye (HemiCy)‐lipophilic triphenylphosphonium cation (TPP) conjugate to develop a mitochondria‐targeted PA probe, named ETP. The phosphate ester linker quenched the PA signal due to the intracellular charge transfer (ICT) process, but can be cleaved by ALP, causing a distinct red‐shift of the ETP to activate the PA signals.^[^ ^70^ ^]^ Using the same phosphate ester linker, Wu et al. designed an activatable PAI probe C^1^X‐OR^1^, which showed a red shifted absorption band at 684 nm when the linker was hydrolyzed and enabled visualization of ALP activity in drug‐induced liver injury.^[^ ^176^ ^]^ In addition to ALP, leucine aminopeptidases (LAP) are also associated with liver disorder. By using a LAP‐responsive leucyl linker, Huang et al. constructed a LAP probe (DLP), which could release the NIR chromophore with red‐shifted absorption to produce a significant photoacoustic signal at 705 nm for multispectral optoacoustic tomography imaging of ALP activity and acetaminophen‐induced liver injury in HepG2 tumor‐bearing mice.^[^ ^177^ ^]^

Other enzyme‐responsive linkers, such as the DEVD linker^[^ ^53^, ^54^ ^]^ and the VC linker,^[^ ^41^ ^]^ have also been applied to construct enzyme‐responsive PAI probes. For example, Fu et al. conjugated nanogapped gold nanoparticles (AuNNPs) with a NIR‐II fluorescent (FL) molecule IR‐1048 using the DEVD linker to produce a PAI probe named AuNNP@DEVD‐IR1048 to image radiotherapy (RT) induced apoptosis. The probe could self‐assemble into AuNNPs aggregates to activate the PA signal at 1250 nm due to the plasmonic coupling effect between the neighboring AuNNPs for early prediction and real‐time evaluation of RT effect.^[^ ^53^ ^]^ In another report, Wang et al. also developed a PA imaging probe (1‐RGD) using the DEVD linker to image chemotherapy induced U87MG tumor cells apoptosis. The cleavage of DEVD linker could induce strong intermolecular interaction and cause the formation of self‐assembled nanoparticles which greatly amplify the PA signal in DOX‐treated tumors compared to saline‐treated tumors (ΔPA≈553 vs. ΔPA≈127) by augmenting nonradiative relaxation of the excited ICG fluorophores within nanoparticles.^[^ ^54^ ^]^ Additionally, to image cathepsin B, a Cypate‐CBT PA probe was developed using the VC linker, the cleavage of which would generate a Cypate‐CBT‐Dimer and produce Cypate‐CBT‐NPs, causing aggregation‐induced PA signal enhancement (Figure 6B).^[^ ^41^ ^]^

#### 3.3.3 pH‐responsive linkers used in PAI

pH‐responsive linkers have also been employed to construct PAI probes for drug release monitoring, tumor imaging and PAI‐guided therapy. For instance, Yang et al. used an acid‐responsive amine‐substituted perdiimide (PDI) derivative, a pH insensitive NIR dye (IR825), and anticancer drug doxorubicin (DOX) to self‐assemble into a diagnostic platforms (THPDINs) for real‐time ratiometric PA imaging of the tumor acidic pH and monitoring drug release in U6MG tumor‐bearing mice. Stimulated by the tumor acidic environment, the pH‐responsive PDI was protonated, followed by the dissociation of the nanostructures, which triggered the release of DOX and disappearance of PA signal at 680 nm. Meanwhile, the chemical structure of IR825 remained unchanged with its characteristic PA signal at 825 nm. Therefore, the DOX release process could be monitored by the ratiometric PA signal at PA_825_/PA_680_ (Figure 6C).^[^ ^132^ ^]^ Yan et al. synthesized a photostable and reversible pH‐responsive phenazine dye (PIOH) based on the pH‐responsive phenazine structure and encapsulated it in liposomes (PIOH‐NPs) for photoacoustic imaging‐guided photothermal therapy in MDR‐231 tumor bearing mice. The PIOH showed a green open‐loop form at 600–850 nm in the tumor acidic environment with strong NIR absorption and good photothermal effect, while it showed a yellow closed‐loop form in the weakly alkaline solution.^[^ ^133^ ^]^

### 3.4 In positron emission tomography (PET) imaging

Positron emission tomography (PET) is a powerful non‐invasive molecular imaging technique that uses radiotracers to real‐timely monitor biomarkers with excellent sensitivity. Contrast‐enhanced PET images are mainly created by selectively retaining radioactivity where the molecular target is present, and a common strategy is based on the binding of radiolabeled ligands to the target receptors. When the molecular target is an enzyme, it is challenging to generate PET imaging contrast.^[^ ^48^ ^]^ To address this issue, a range of enzyme‐responsive linkers have been developed to design PET tracers to precise image enzyme activity in tumors and diagnosing enzyme‐related diseases.

As an important regulator of apoptosis, caspase‐3 represents an important molecular target, and the imaging of which is of great significance for evaluation of early therapeutic efficacy. Detection of apoptosis with PET has been achieved by using capase‐3‐resposive linkers resulting in an advantageous sensitivity. For instance, Xia et al. developed a[^18^F]‐CP18 probe using the DEVD linker which could be cleaved by activated caspase‐3 and subsequently cause accumulation of polar DEVD [^18^F]‐radiolabeled metabolite in the cytoplasm of apoptotic cells, leading to enhanced ^18^F activity.^[^ ^55^ ^]^ Similarly, Qiu et al. designed a PET probe [^18^F] DEVD‐Cys(StBu)‐PPG(CBT)‐AmBF_3_ ([^18^F] I) which was able to self‐assembled into nanoparticles to generate ^18^F activity in situ upon caspase cleavage, with a 2.2 folds more retained ^18^F in Dox treated apoptotic HeLa cells compared to the control cells, indicating the great promise of the caspase‐responsive linker for PET imaging of drug‐induced apoptosis.^[^ ^56^ ^]^

In addition to caspase, linkers that can respond to legumain, a biomarker overexpressed in several cancers such as breast cancer, gastric cancer, colorectal cancer, have been developed for PET imaging of legumain activity and diagnosis of legumain‐related diseases. For example, Qiu et al. employed an Ala‐Ala‐Asn (AAN) linker to conjugate cyanobenzothiazole and cysteine moieties to synthesize a PET tracer ^18^F‐2. Upon cleavage by legumain, the cyanobenzothizole and cysteine could self‐assemble into nanoparticles which enhanced the accumulation and retention of radioactivity in HCT116 tumors, with an improved tumor‐to‐background contrast.^[^ ^178^ ^]^ Lu et al. synthesized a new PET tracer [^18^F]SF‐AAN also using the AAN linker, the cleavage of which would lead to exposure of the sulfhydryl and amino group of cysteine to condensate with 2‐cyano group of 6‐amino‐2‐cyanobenzothiazole (CBT) and formation of an intramolecular cyclized compound [^18^F]SF‐C. The self‐assembled [^18^F]SF‐C aggregates were able to generate a strong radioactive signal for PET imaging of legumain activity in MDA‐MB‐468 tumors with a high sensitivity.^[^ ^179^ ^]^

**FIGURE 7:**
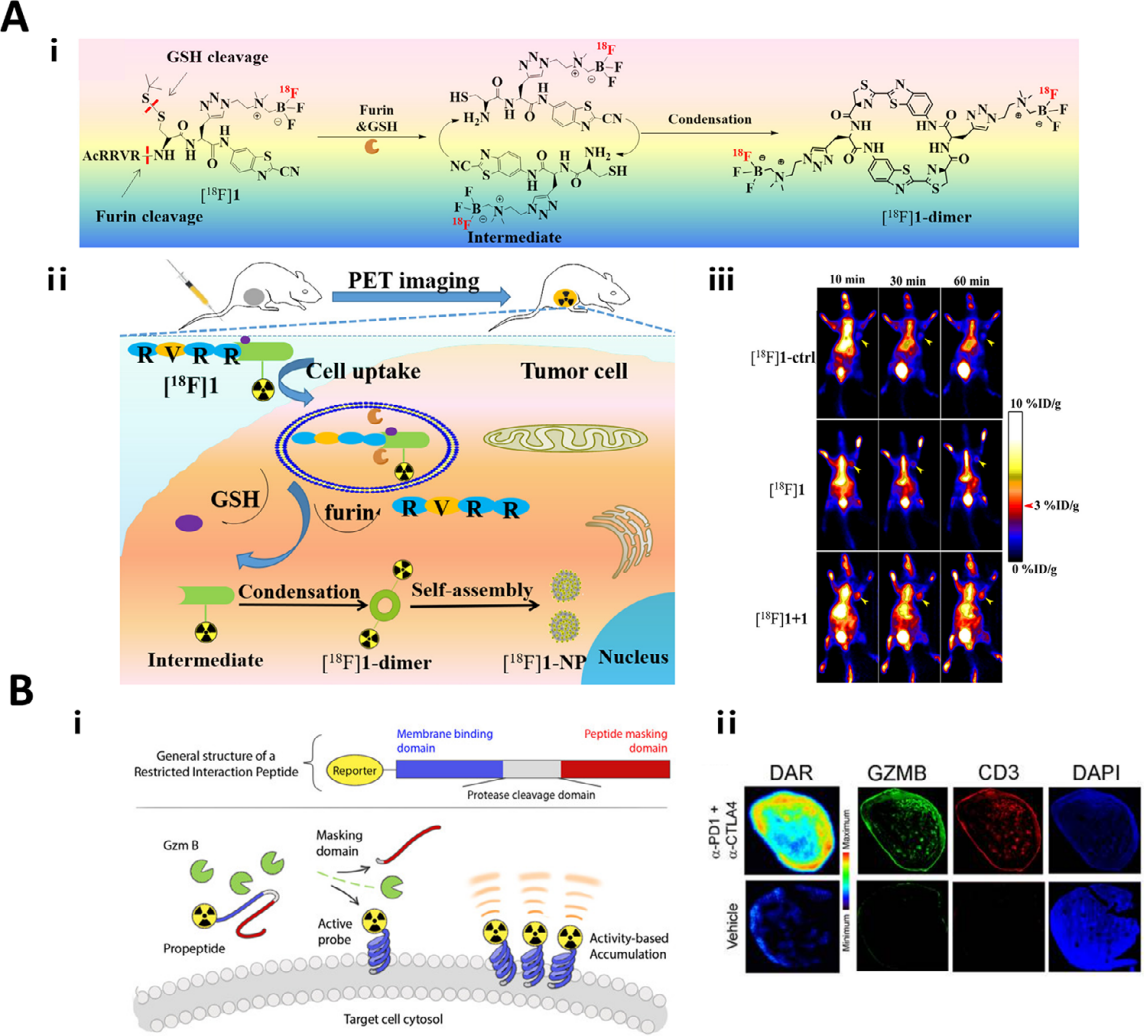
Responsive linkers for positron emission tomography imaging. A) Furin‐responsive linker used for tumor imaging. Condensation of [^18^F]1 after cleaved by furin and GSH; Self‐assembly of [^18^F]1 in tumor cells; In vivo tumor imaging of [^18^F]1. The self‐assembly of [^18^F]1 in tumors could be enhanced by the co‐injection with the non‐radioactive compound 1 owing to that the non‐radioactive compound could help the furin‐controlled intracellular condensation and self‐assembly of [^18^F]1 into [^18^F]1‐NPs in tumor cells. Reproduced with permission.^[^ ^68^ ^]^ Copyright 2020, Elsevier Inc. B) Granzyme B‐responsive linker used for monitoring granzyme B activity. Cleavage of the full‐length pro‐form by a dedicated endoprotease liberates a radiolabeled antimicrobial peptide that can accumulate to reflect the relative units of enzyme activity in a region of interest; Digital autoradiography and immunofluorescence showing the colocalization of ^64^Cu‐GRIP B with GZMB and T cells within CT26 tumor slices from mice exposed to a vehicle or CPI. Reproduced with permission.^[^ ^28^ ^]^ Copyright 2021, American Chemical Society.

Another example of developing activatable PET imaging probes takes advantage of furin. For instance, Liang et al. synthesized a RVRR linker that could be cleaved by overexpressed furin in MDA‐MB‐468 tumors, and used it to construct a PET tracer, CBT‐^68^Ga. The cleavage of RVRR linker could generate amphiphilic oligomers (CBT‐Ga‐Dimer and CBT‐Ga‐Trimer) which would self‐assemble into nanoparticles CBT‐Ga‐NPs, resulting in prolonged radioactivity retention in MDA‐MB‐468 cancer cells and an 9.1‐fold signal enhancement of tumor/liver ratio compared to the control.^[^ ^67^ ^]^ In addition, by using the RVRR linker, the same group also designed a ^18^F‐based PET tracer, [^18^F]1, which was able to spontaneously condensate between the intermediates to produce a rigid and lipophilic dimer ([^18^F]1‐dimers) responding to the furin activity. As a result, the probe could further be self‐assembled into nanoparticles ([^18^F]1) in tumor cells, improving the efficiency and accuracy of PET imaging of furin‐overexpressing MDA‐MB‐468 tumors (Figure 7A).^[^ ^68^ ^]^

Enzyme cleavable linkers can also be employed for PET imaging of immune activities. Recently, granzyme‐B responsive linkers have been developed for PET imaging of granzyme B activity and further used for monitoring tumor response to immunotherapy. For example, Zhao et al. used a granzyme‐B responsive IEPDVSVQ linker to develop a PET imaging probe ^64^Cu‐labeled GRIP B. The imaging probe ^64^Cu‐labeled GRIP B was consisted of three domains: (1) a nontoxic antimicrobial peptide (AMP) coupled to a radioisotope, (2) a specific granzyme B cleavage site, and (3) a peptide “masking” domain that prevents the AMP from adopting its preferred helical conformation that tightly binds phospholipid bilayers. The cleavage of IEPDVSVQ linker by granzyme B released a radiolabeled form of Temporin L, which sequestered radioisotope through binding to adjacent tumor cell phospholipid bilayers, whose tissue biodistribution in the body reflected the relative unit of granzyme B activity, hence realizing measurement of granzyme B and T cells activation with immune checkpoint inhibitors (CPI) (Figure 7B).^[^ ^28^ ^]^

## 4 CONCLUSION AND PROSPECT

As a new paradigm for the accurate diagnosis of diseases, precise imaging of tumors, and monitoring treatment efficacy of cancers, activatable imaging probes based on responsive linkers present numerous benefits over traditional imaging methods, such as higher imaging SBR values, real‐time imaging capacity, excellent sensitivity, as well as precise diagnosis of diseases at molecular level. In this review, we have summarized the types of responsive linkers and their biomedical applications in molecular imaging, including optical imaging, MRI, PAI, and PET imaging, providing a reference for linkers' research in the development of highly sensitive molecular imaging probes. Optical imaging has added an easy and economical modality to the rapidly growing field of molecular imaging. We concluded that the design principle activatable optical imaging probes are usually composed of two or three basic parts: i) stimuli‐linker which can be recognized and cleaved, ii) moiety matched with a fluorophore which generates a strongly activated signal after substrate cleavage, iii) special part that optimizes pharmacokinetics or improves the cellular uptake of the probe.

Despite the great advancements of responsive linkers in the field of molecular imaging, there are still some challenges that need to be addressed for accelerating their clinical translation. For instance, 1) the potential toxicity of these linkers has been overlooked in almost all studies cited in this review. Safety evaluation on them and their cleaved forms require more in‐depth research, including their pharmacokinetics, biodistribution, biodegradability etc., which are critical for their clinical application. 2) Another important concern about responsive linkers is their sensitivity to stimuli and effective cleavage at target sites to achieve accurate and sensitive molecular imaging. Due to the heterogeneity of tumors (either different stages of tumors or differences among the various tumors), as well as the differences between various organisms, disease‐related biomarkers may have varying levels of expression in organisms, which may lead to non‐specific cleavage or insufficient cleavage of the linkers at target sites, resulting in the ineffective release of cargos and hence reducing imaging sensitivity and diagnostic efficiency. Therefore, improving the stimuli response function of linkers at the target sites needs to be further explored to improve the imaging sensitivity and diagnostic efficiency. Regarding the enhancement of sensitivity, the efficacy of linkers is related with the endogenous concentrations of different stimuli. In these cases, the stimuli levels could be enhanced artificially. For example, the use of light to produce singlet oxygen by means of photosensitizers has been used to provide higher ROS concentrations combined with ROS‐responsive linkers to improve the sensing sensitivity.^[^ ^180^ ^]^ Modifying sequences that can react faster and more specifically with biomarkers^[^ ^28^, ^71^ ^]^ or can improve cell permeability^[^ ^39^ ^]^ on linkers to increase concentration in target cells is a promising strategy for increasing adequate and specific cleavage of linkers. In addition, the dual‐locked strategy utilizing two disease‐related biomarkers as triggers for linker cleavage, which can also improve the specificity and accuracy of imaging and diagnosis.^[^ ^29^ ^]^

Although there are many obstacles in the way of clinical translation, we think that the ingenious design and construction of responsive linkers for molecular imaging is an essential area of interest for disease diagnosis and treatment detection. Along with detecting disease‐related biomarkers and molecular imaging, other interesting technological uses of linkers have emerged. Among them, responsive linkers have also been used to control the opening of drug‐release gates, or to change the characteristics or the conformation of nanoparticles upon exposure.^[^ ^181^ ^]^ It is our hope that, with the information provided in this review, more potent responsive linkers with great performance will soon be available for the development of molecular imaging probes and precise diagnosis of cancer or other diseases in the future.

## CONFLICT OF INTEREST STATEMENT

The authors declare no conflicts of interest.
